# TIMELESS Promotes LUAD Growth via Suppressing Transferrin-Mediated Ferroptosis and Reprograms the Tumor Microenvironment against Anti-PD-1 Immunotherapy

**DOI:** 10.34133/cancomm.0009

**Published:** 2026-02-03

**Authors:** Chenchen Hu, Feiming Hu, Changjian Shao, Yuanli He, Liping Su, Daimei Shi, Lingying Yu, Yuanjie Sun, Jing Wang, Xiyang Zhang, Hongtao Duan, Junqi Zhang, Yubo Sun, Dongbo Jiang, Xiaolong Yan, Shuya Yang, Kun Yang

**Affiliations:** ^1^ Department of Immunology, Basic Medicine School, Air Force Medical University, Xi’an 710032, Shaanxi, P. R. China.; ^2^ Department of Thoracic Surgery, Tangdu Hospital, Air Force Medical University, Xi’an 710038, Shaanxi, P. R. China.; ^3^ Military Medical Innovation Center, Air Force Medical University, Xi’an 710032, Shaanxi, P. R. China.

## Abstract

**Background:** Lung cancer remains a major global health burden. RNA-binding proteins (RBPs) play crucial roles in post-transcriptional gene regulation, and their dysregulation is frequently implicated in tumorigenesis. The present study aimed to elucidate the molecular network governed by the highly expressed RBP TIMELESS in lung adenocarcinoma (LUAD) and determine its mechanistic role in LUAD progression. **Methods:** The Cancer Genome Atlas-LUAD, Gene Expression Omnibus, and single-cell RNA sequencing datasets were analyzed to identify aberrantly expressed RBP genes. The RBP gene *TIMELESS* exhibited the most significant effect on LUAD cell death and was selected for further study. Photoactivatable ribonucleoside-enhanced crosslinking and immunoprecipitation sequencing and RNA sequencing were employed to identify ferroptosis-related targets directly bound by TIMELESS. Molecular mechanisms underlying the TIMELESS-mediated regulation of ferroptosis in LUAD were investigated via immunoprecipitation–mass spectrometry, glutathione *S*-transferase pull-down, immunofluorescence–fluorescence in situ hybridization, RNA immunoprecipitation, poly(A)-tail, and RNA stability assays. In an orthotopic lung cancer mouse model treated with erastin (a ferroptosis inducer) and programmed cell death protein 1 (PD-1) blockade, the role of TIMELESS in therapeutic response was assessed via flow cytometry and multiplex immunofluorescence (mIF). Infiltrating immune cells in LUAD were analyzed by tissue microarrays (TMAs) via mIF. **Results:** TIMELESS significantly affected LUAD cell proliferation and death, and *TIMELESS* knockdown significantly enriched RNA-binding and ferroptosis pathways. Transferrin (TF) was identified as a direct TIMELESS target governing ferroptosis. TIMELESS was revealed to bind Ccr4-Not transcription complex subunit 3 (CNOT3) to promote TF mRNA degradation. TIMELESS depletion combined with erastin and PD-1 blockade enhances efficacy, prolongs survival, increases T cell and M1 macrophage infiltration, and reduces M2 macrophage infiltration. Further, high TIMELESS expression was inversely correlated with ferroptosis marker 4-hydroxynonenal but positively correlated with programmed cell death ligand 1 (PD-L1), reduced T cell and M1 macrophage infiltration, and increased M2 macrophage infiltration. **Conclusions:** TIMELESS recruits CNOT3 to accelerate TF mRNA degradation, thereby suppressing ferroptosis and promoting LUAD growth. These findings suggest that the TIMELESS/TF regulatory axis may be a promising therapeutic target for LUAD.

## Background

RNA-binding proteins (RBPs) orchestrate transcriptome-wide regulation by controlling RNA metabolism at transcriptional, post-transcriptional, and translational levels [1]. As master regulators of cellular homeostasis [2,3], RBP dysregulation disrupts transcriptomic balance in cancer, driving oncogenic signaling through aberrant expression of tumor-associated targets [4]. In lung carcinogenesis, RBPs function as pivotal effectors that spatiotemporally reprogram RNA metabolism, immune checkpoint expression, and metabolic pathways, thereby rewiring oncogenic networks and sculpting immunosuppressive tumor microenvironment (TME) [5]. Systematic characterization of RBP networks is thus critical for deciphering cancer cell-intrinsic mechanisms and therapeutic vulnerabilities.

Lung cancer is the leading global cause of cancer mortality, with approximately 1.8 million deaths in 2022 [6–9]. Non-small cell lung cancer (NSCLC) represents 85% of cases, of which lung adenocarcinoma (LUAD) accounts for 40% [10]. LUAD exhibits a distinct etiology, frequently occurring in nonsmoking females with genetic susceptibility [11]. The profound molecular heterogeneity of LUAD, characterized by recurrent driver mutations and genomic instability, underlies variable treatment responses and acquired resistance [12,13]. Although programmed cell death protein 1 (PD-1)/programmed cell death ligand 1 (PD-L1) blockade improves overall survival compared to chemotherapy, most advanced LUAD patients develop resistance, resulting in disease progression [14–16]. Longitudinal analysis of paired tumor specimens pre- and post-immune checkpoint inhibitor (ICI) resistance acquisition has revealed decreased frequencies of resistance-associated mutations, including serine/threonine kinase 11, mammalian target of rapamycin, and kelch-like ECH-associated protein 1, reduced tumor-infiltrating lymphocytes, and diminished human leukocyte antigen class I expression in post-progression biopsies [17]. These findings suggest that immunotherapy induces inflammatory oncogenic mutation acquisition and tumor progression, highlighting the intricate interplay of dynamic mechanisms within the TME [12,18].

The immunosuppressive NSCLC TME enables immune evasion and progression [19]. Spatial transcriptomics has revealed immune-niche heterogeneity predictive of LUAD outcomes [20], where malignant epithelial cells engage in reciprocal crosstalk with stromal and immune compartments [18,21]. Current therapeutic strategies prioritize targeting clonal evolution to predict treatment efficacy, while TME-modulating approaches are in preclinical development. Emerging evidence reinforces promising candidates for cancer immunotherapy, such as anti-CD47 antibodies that enhance macrophage-mediated phagocytosis of tumor cells by disrupting the CD47/signal regulatory protein alpha (SIRPα) axis [22], and C-X-C motif chemokine ligand 10 (CXCL10) agonists that facilitate natural killer (NK) cells infiltration through cytokine chemotactic regulation [23]. Consequently, understanding oncogene–TME interplay is essential for next-generation therapies.

Metabolic symbiosis in the LUAD TME involves RBP-mediated coordination of lactate shuttling, glutaminolysis, and lipid clearance, reshaping stromal and immune cell phenotypes [19]. Ferroptosis, an iron-dependent form of regulated cell death governed by redox imbalance, serves as a critical nexus between tumor metabolism and immunobiology [24,25]. RBPs extensively modulate ferroptosis. YTH domain-containing protein 1 (YTHDC1) post-transcriptionally regulates the ferroptosis suppressor protein 1 (FSP1) to promote ferroptosis in lung cancer cells, thereby suppressing tumor progression [26]. Ferroptosis induction alters TME dynamics mainly through immunogenic cell death to enhance antigen presentation, lipid peroxide accumulation to regulate myeloid cell polarization, and redox imbalance to destroy immunosuppressive metabolites [27,28]. However, RBP-mediated regulation of ferroptosis signaling and its immunotherapeutic implications in LUAD remain incompletely characterized.

The circadian gene *TIMELESS* orchestrates the integration of circadian rhythms and cell cycle progression, serving as a crucial guardian of replication fidelity and DNA damage response [29,30]. Beyond its circadian functions, TIMELESS exhibits oncogenic properties across multiple malignancies, including colorectal cancer [31] and breast cancer [32]. To elucidate the role and mechanism of TIMELESS in LUAD, we conducted an integrated analysis based on clinical data, along with in vitro and in vivo functional assays. This study aimed to clarify the clinical relevance of TIMELESS in LUAD and the molecular mechanisms underlying TIMELESS-mediated regulation of ferroptosis and the tumor immune microenvironment.

## Methods

### Cell culture

The human LUAD cell lines A549 and NCI-H1975 (H1975), human normal bronchial epithelial cell line BEAS-2B, and mouse Lewis lung carcinoma cell line LLC1 were cultured in RPMI 1640 medium (Gibco, Cat. #11875093) supplemented with 10% fetal bovine serum (FBS; Gibco, Cat. #10270) and 1% streptomycin–penicillin solution (Solarbio, Cat. #P1400) at 37 °C under 5% CO_2_. All cell lines used in this study were tested mycoplasma-negative and authenticated by short-tandem repeat analysis.

### Sample collection

Two human LUAD tissue microarrays (TMAs) containing both tumor and adjacent normal tissues were obtained from Shanghai Outdo Biotech Co., Ltd., encompassing 90 and 92 patient cases for TMA 1 and TMA 2, respectively. Clinicopathological data for these TMAs are provided in Tables S1 and S2. After initial staining, 87 tumor samples from TMA 1 remained analyzable and were designated as cohort 1; their characteristics stratified by TIMELESS or TF expression levels are summarized in Table 1. TMA 2, designated as cohort 2, was subjected to multiplex immunofluorescence (mIF). After multiple staining rounds, 38 matched tumor–normal pairs yielded complete data for all markers (TIMELESS, 4-hydroxynonenal [4HNE], PD-L1, CK7, CD4, CD8, CD86, and CD206). Furthermore, TIMELESS and PD-L1 staining data were available for an additional 42 tumor samples with associated survival information.

**Table 1.** Associations between TIMELESS and TF expression and clinicopathological parameters in LUAD patients. Patients were classified into high and low TIMELESS and TF expression groups based on the median expression value.

Characteristic	Whole cohort [*n* (%)]	TIMELESS expression [*n* (%)]		*χ* ^2^	*P*	TF expression [*n* (%)]		*χ* ^2^	*P*
		Low	High			Low	High		
**Total**	87 (100.00)	44 (100.00)	43 (100.00)			44 (100.00)	43 (100.00)		
**Gender**				0.946	0.331			0.099	0.752
Male	46 (52.87)	21 (47.73)	25 (58.14)			24 (54.55)	22 (51.16)		
Female	41 (47.13)	23 (52.27)	18 (41.86)			20 (45.45)	21 (48.84)		
**Age**				2.105	0.149			3.105	0.078
>65 years	28 (32.18)	11 (25.00)	17 (39.53)			18 (40.91)	10 (23.26)		
≤65 years	59 (67.82)	33 (75.00)	26 (60.47)			26 (59.09)	33 (76.74)		
**Tumor size**				5.090	0.024			4.274	0.039
>3 cm	38 (43.68)	14 (31.82)	24 (55.81)			24 (54.55)	14 (32.56)		
≤3 cm	49 (56.32)	30 (68.18)	19 (44.19)			20 (45.45)	29 (67.44)		
**AJCC stage**				4.398	0.036			8.996	0.003
I–II	67 (77.01)	38 (86.36)	29 (67.44)			28 (63.64)	39 (90.70)		
III	20 (22.99)	6 (13.64)	14 (32.56)			16 (36.36)	4 (9.30)		
**N stage**				0.811	0.368			3.756	0.053
N0–1	72 (82.76)	38 (86.36)	34 (79.07)			33 (75.00)	39 (90.70)		
N2–3	15 (17.24)	6 (13.64)	9 (20.93)			11 (25.00)	4 (9.30)		
**T stage**				7.441	0.006			7.026	0.008
T1–2	77 (88.51)	43 (97.73)	34 (79.07)			35 (79.55)	42 (97.67)		
T3–4	10 (11.49)	1 (2.27)	9 (20.93)			9 (20.45)	1 (2.33)		

AJCC, American Joint Committee on Cancer; LUAD, lung adenocarcinoma; TF, transferrin

Patient data and tumor tissues were collected with informed consent. Clinical data, including tumor staging and treatment history, were retrieved from medical records. All samples were histologically confirmed as LUAD prior to analysis. Additionally, 10 paired human LUAD and adjacent normal tissue samples (cohort 3) were collected between 2022 and 2024 from Tangdu Hospital, Air Force Medical University, for immunohistochemistry (IHC) staining and organoid culture. The present study complied with all relevant ethical regulations governing human participant research. The Air Force Medical University Institutional Review Board approved human sample collection (No. KY20234063-1).

### TCGA-LUAD and GEO data analysis

A total of 2,961 RBPs were obtained from the published datasets and the EuRBPDB database [33–35]. The integrated catalog, presented in Table S3, comprises 1,826 canonical and 1,135 noncanonical RBPs. Differential expression analysis of RBP genes was performed between LUAD and adjacent normal tissues using RNA sequencing (RNA-seq) data from The Cancer Genome Atlas (TCGA)-LUAD cohort and data from 4 Gene Expression Omnibus (GEO) datasets (GSE40419, GSE32863, GSE75037, and GSE253013). Based on the TCGA-LUAD data, Kaplan–Meier survival analysis and univariate Cox proportional hazards regression analysis were conducted to evaluate the prognostic significance of RBP expression. Patients were stratified into high- and low-expression groups using the median expression value of each RBP gene as the cutoff. Analysis of TIMELESS expression in cancer cells and the proportion of main cell types in LUAD patients pre- and post-treatment was performed using a single-cell RNA sequencing (scRNA-seq) dataset (GSE207422). Immune cell infiltration levels in TCGA-LUAD samples were quantified using the ImmuCellAI database [36]. Spearman’s rank correlation was applied to assess the correlation between TIMELESS expression and the degree of immune cell infiltration.

### Generation of CRISPR/Cas9 knockout tumor cell lines

A549 and H1975 *TIMELESS* knockout cell lines were generated using the CRISPR/Cas9 system (GenePharma, Shanghai, P. R. China). For the construction of single-guide RNA (sgRNA) expression plasmids, complementary DNA oligonucleotides were annealed and bridged to the LentiV2-gRNA/Cas9/Puro lentiviral vectors. Cells were subsequently transfected with LentiV2-gRNA/Cas9/Puro vectors carrying the target sgRNAs. Three days post-puromycin selection, surviving cells were seeded into 96-well plates at limiting dilution for single-cell cloning. Individual clones were isolated after 15 days, and the efficiency of *TIMELESS* gene editing was validated by quantitative real-time polymerase chain reaction (qPCR) and Western blotting. Selected *TIMELESS*-knockout clones were expanded and designated as sg*TIMELESS* cells. Control cells transfected with nontargeting sgRNA vectors were designated sg*Ctrl* cells. The sequences of siRNAs, sh/sgRNAs, and plasmids are shown in Table S4.

### Reverse transcription quantitative real-time PCR, RNA stability analyses, poly(A) tail assay, and RNA-seq

To quantify gene expression, total RNA was extracted from *sgTIMELESS* and *sgCtrl* LUAD cells using an RNA extraction kit (Tsingke, Cat. TSP413) and reverse transcribed into cDNA. Reverse transcription qPCR (RT-qPCR) was performed using SYBR Green PCR Master Mix (TaKaRa, Cat. RR037A). To assess the RNA stabilities of TF and Ccr4-Not transcription complex subunit 3 (CNOT3), H1975 and A549 cells were treated with 2.5 μmol/l actinomycin D (MedChemExpress, Cat. HY-17559) to inhibit transcription. RNA was harvested at the indicated time points (0, 1, 2, 4, 6, 8, and 10 h), and the remaining transcript levels were determined by RT-qPCR. Poly(A) tail (PAT) assay was performed as described previously [37], and the PAT length was analyzed by PCR. The primers used in the above experiments were purchased from Tsingke Biotech Co., Ltd., and the primer sequences are shown in Tables S5 and S6.

For RNA-seq analysis, total RNA was extracted from H1975 cells with knockdown of *TIMELESS*, centromere protein F (*CENPF*), kinesin family member 20A (*KIF20A*), or protein regulator of cytokinesis 1 (*PRC1*); control cells were processed in parallel. RNA samples were submitted to LC-Bio Technology Co., Ltd. for library preparation and paired-end sequencing to identify differentially expressed genes. Functional enrichment analysis of these genes was performed using the “clusterprofiler” package for Kyoto Encyclopedia of Genes and Genomes (KEGG), Gene Ontology (GO), and Reactome terms, with pathways meeting a significance threshold of *P* < 0.05 being presented.

### Western blotting, immunoprecipitation, and immunoprecipitation–mass spectrometry

Protein expression was assessed by Western blotting as previously described [38]. The antibodies used for Western blotting are listed in Table S7.

Total protein was extracted from H1975 cells using lysis buffer supplemented with protease and phosphatase inhibitors. For immunoprecipitation (IP), cell lysates were incubated overnight at 4 °C with anti-TIMELESS antibody, anti-CNOT3 antibody, or anti-IgG isotype control antibody, together with Protein A/G PLUS-Agarose beads (Santa, Cat. sc-2003). The beads were then washed 3 times with lysis buffer for subsequent Western blotting analysis. For IP-mass spectrometry (IP-MS) analysis, the immunoprecipitated complexes were resolved by sodium dodecyl sulfate polyacrylamide gel electrophoresis. Proteins within the gel bands were subjected to in-gel tryptic digestion, and the resulting peptides were analyzed by MS to identify the proteins interacting with the target protein.

Flag-tagged TIMELESS mutants targeting key residues (R940A, K930A, R926A, Q898A, and V887A) were synthesized and sequence-verified by Tsingke Biotech Co., Ltd. Plasmids were transfected into H1975 cells using Lipofectamine 3000 (Thermo, Cat. L3000015). After 48 h, cell lysates were immunoprecipitated using anti-Flag magnetic beads at 4 °C overnight. Eluted proteins were analyzed by immunoblotting with an anti-CNOT3 antibody.

### Cell counting kit-8 and half-maximal inhibitory concentration determination

Cells (5 × 10^3^ per well) were seeded in 96-well plates and incubated overnight. Erastin (MedChemExpress, Cat. HY-15763) or RSL3 (MedChemExpress, Cat. HY-100218A) was then added to the wells at various concentrations (0, 0.1, 1, 3, 5, and 10 μmol/l) for 48 h. After incubation, the culture medium was discarded. Cell counting kit-8 (CCK-8) reagent (MedChemExpress, Cat. HY-K0301) was added to each well, and the absorbance at 450 nm was measured using a microplate reader (TECAN, Infinite 200 PRO). The half-maximal inhibitory concentration (IC_50_) value was calculated according to the previous method [39].

### Calcein-AM/PI staining

The proportion of live and dead cells was detected using a Calcein/PI cell viability assay kit (Beyotime, Cat. C2015M), according to the manufacturer’s instructions. To assess the effect of ferroptosis inhibition, *sgTIMELESS* and *sgCtrl* LUAD cells were treated with 2 μmol/l ferrostatin-1 (MedChemExpress, Cat. HY-100579) for 48 h prior to the assay. LUAD cells subjected to different treatments were collected. The Calcein-acetoxymethyl (AM) and propidium iodide (PI) working fluid was added to the cells, followed by incubation at 37 °C for 30 min in the dark. The stained cells were analyzed by flow cytometry to determine the proportions of live (Calcein-AM positive) and dead (PI positive) cells.

### Lipid peroxidation malondialdehyde assay

The relative intracellular malondialdehyde (MDA) concentration was measured using a lipid peroxidation MDA assay kit (Abcam, Cat. ab118970). Briefly, MDA produced in the treated cells reacted with thiobarbituric acid (TBA) solution to generate red MDA-TBA adducts. The absorbance of MDA-TBA adduct was detected at 532 nm by colorimetry, and results were normalized to protein concentrations.

### Measurement of cellular and lipid reactive oxygen species, ferroptosis-related lipid peroxidation, and mitochondrial membrane potential

*TIMELESS*-knockout LUAD cells were seeded in a 6-well plate and treated with dimethyl sulfoxide (DMSO), erastin (3 μmol/l for H1975 and 5 μmol/l for A549, respectively), or RSL3 (0.7 μmol/l for H1975 and 1.1 μmol/l for A549, respectively) at concentrations specific to each cell line. Cells were then washed with phosphate-buffered saline (PBS) and incubated in serum-free medium with the following fluorescent probes at 37 °C for 30 min: 10 μmol/l DCFH-DA (Beyotime, Cat. S0033S) for total reactive oxygen species (ROS), 1 mmol/l Liperfluo (DOJINDO, Cat. L248) for lipid peroxides, and 5 μmol/l BODIPY 581/591 C11 (Invitrogen, Cat. D3861) for lipid peroxidation. For flow cytometry analysis, 30,000 single cells per sample were assessed. The red-to-green fluorescence ratio of BODIPY 581/591 C11 levels reflects the relative levels of oxidized and reduced lipids, serving as an indicator of lipid peroxidation.

Mitochondrial membrane potential (MMP) was evaluated using the JC-1 mitochondrial membrane potential assay kit (Yeasen Biotechnology, Cat. 40705ES03) according to the instructions. In brief, treated cells cultured in 6-well plates were stained with 5× JC-1 staining solution at 37 °C in the dark, followed by 2 washes with the kit-provided buffer. After 20 min, cells were washed twice with 1× staining buffer and analyzed by flow cytometry. The red/green fluorescence ratio reflected MMP changes.

### Assessment of mitochondrial mass, membrane potential, and superoxide production

Mitochondrial parameters were evaluated in H1975 cells using a multiplexed fluorescence staining approach. Cells were first washed with Hank’s Balanced Salt Solution (HBSS) and then incubated for 30 min at 37 °C in 5% CO₂ with a cocktail containing 1 μg/ml Hoechst 33342 for nuclear counterstaining, 100 nmol/l MitoTracker Green FM for mitochondrial mass quantification, and 150 nmol/l tetramethylrhodamine ethyl ester (TMRE) for assessing MMP. Following incubation and 2 washes with HBSS, cells were subsequently stained with 10 μmol/l MitoSOX Deep Red reagent for 30 min at 37 °C to detect mitochondrial superoxide generation. After final washing, multichannel images were acquired using a confocal laser scanning microscope (Olympus, FV3000).

### Cell and tissue iron measurement

Total cellular iron was measured by Cell Total Iron Colorimetric Assay Kit (Elabscience, Cat. E-BC-K880-M). Approximately 1 × 10^6^ cells were lysed and mixed with the detection reagent at 37 °C for 40 min. Optical density‌ values were measured at a wavelength of 593 nm using a microplate reader (TECAN, Infinite 200 PRO). Intracellular Fe^2+^ was stained in live cells with FerroOrange fluorescent probe (DOJINDO, Cat. F374). Cells were incubated with 1 μmol/l FerroOrange for 20 min at 37 °C. Images were captured by a confocal laser scanning microscope (Olympus, FV3000) at 561 nm excitation and 600 nm emission.

Total and ferrous iron in lung tumor tissues were detected by Total Iron Colorimetric Assay Kit and Ferrous Iron Colorimetric Assay Kit (Elabscience, Cat. E-BC-K772 and K773). Fresh tissue (0.1 g) was homogenized in the detection reagent. After centrifugation, the absorbance of the supernatant was measured at 593 nm. The iron concentration in each sample was calculated from the measured absorbance according to the kit’s instructions.

### Photoactivatable ribonucleoside-enhanced crosslinking and IP and RNA immunoprecipitation

Photoactivatable ribonucleoside-enhanced crosslinking and immunoprecipitation (PAR-CLIP) was performed as previously described [40]. The experiment was conducted in H1975 cells using an antibody against TIMELESS, with normal rabbit IgG serving as the negative control. Specifically, cells were incubated with 4-thiouridine (100 μmol/l) for 16 h, crosslinked with 365-nm ultraviolet light on ice, and then subjected to IP. The isolated complexes were treated with RNase, and the bound RNAs were finally released by proteinase K digestion for high-throughput sequencing.

RNA immunoprecipitation (RIP) was conducted according to established methods [40]. Lysates from H1975 and A549 cells cultured in 150-mm culture dishes were immunoprecipitated with anti-TIMELESS or anti-IgG antibody. Coprecipitated RNA was reverse transcribed and quantified by RT-qPCR.

### Immunofluorescence with fluorescent in situ hybridization and immunofluorescence staining

Cells in the confocal dishes were fixed with paraformaldehyde (PFA), digested with protease K, and then hybridized with FAM488-TF probe. After washing and blocking, samples were incubated with an anti-TIMELESS antibody overnight at 4 °C, followed by a 50-min incubation with a Cy3-labeled goat anti-rabbit secondary antibody at room temperature. 4',6-Diamidino-2-phenylindole dihydrochloride (DAPI) was used to counterstain the nucleus for confocal imaging.

For immunofluorescence (IF) staining, samples were sequentially incubated with primary antibodies against TF and CNOT3, followed by corresponding fluorescent secondary antibodies. Nuclei were counterstained with DAPI, and images were acquired using a confocal laser scanning microscope (Olympus, FV3000). Detailed antibody information is provided in Table S7.

### Luciferase reporter assay

The transcriptional activity of the TF promoter was assessed using a Dual Luciferase Reporter System (Promega, Cat. E1960). H1975 and A549 cells at 60% to 80% confluence were transfected with reporter plasmids carrying either the wild-type (WT) or mutant (MUT) TF promoter sequence, using Lipofectamine 3000. For the 3′UTR reporter assay, cells in 24-well plates were cotransfected with either the PGL3-TF-3′UTR-WT or PGL3-TF-3′UTR-MUT reporter plasmid, along with a TIMELESS overexpression plasmid. Luciferase activity was measured in cell lysates 48 h post-transfection. All plasmid sequences are detailed in Table S8.

### Glutathione *S*-transferase pull-down assays

A glutathione *S*-transferase (GST) pull-down assay was conducted according to the GST Pull-Down Kit (FITGENE, Cat. FI8804) instructions. About 50 μl of *Escherichia coli* expressing GST-TIMELESS and His-CNOT3 was collected by centrifugation, washed with PBS, and resuspended in 500 μl of pre-cooled lysis buffer containing protease inhibitors. The post-sonication supernatant was incubated with glutathione resin pre-loaded with GST-TIMELESS or GST empty vector. After 2 h of binding at 4 °C with rotation, the resin was centrifuged, washed, and incubated at 4 °C overnight with His-CNOT3-containing lysate. Western blotting analysis was performed on eluted proteins after washing. The sequences for the GST-TIMELESS and His-CNOT3 constructs are detailed in Table S8.

### Transmission electron microscope

Samples from cells and lung tumor tissues underwent primary fixation with a standard electron microscopy fixative, 2.5% glutaraldehyde. After washing with 0.1 mol/l phosphate buffer (PB, pH 7.4), they were subjected to post-fixation with 1% osmium tetroxide in PB (pH 7.4) to stabilize lipids and enhance contrast. Samples were dehydrated in a graded ethanol series, infiltrated twice in 100% acetone (15 min each). After overnight incubation in the oven at 37 °C, the sample was embedded in SPI-Pon812 (hedebio, Cat. SPI-02660) and polymerized in the oven at 60 °C for 48 h. Ultrathin sections (60 to 80 nm thick) were cut using an ultramicrotome, mounted on formvar/carbon-coated copper grids, and visualized by a transmission electron microscope (TEM).

### Tissue processing

Patient tissues were collected in ice-cold RPMI 1640 medium and transferred to a biosafety cabinet. After washing with RPMI 1640, the nontumor muscle and fat were excised. Fresh tumor tissues were divided into 3 portions. One portion was minced and filtered through a 100-μm cell strainer for organoid derivation. Another portion was fixed in 4% PFA for histopathological analysis, and the remaining tissue was stored in liquid nitrogen.

### Patient-derived organoids culture

The Air Force Medical University Institutional Review Board approved sample collection (No. KY20234063-1). Primary tumor tissues were processed using the Human Lung Cancer Organoid Culture Medium Kit (Absin, Cat. abs9443). Tissue was digested with human lung cancer primary tissue digestion fluid C and incubated at 37 °C with agitation for 10 to 20 min. Resulting cells were collected by centrifugation at 300 ×*g* for 5 min, resuspended in Matrigel (Absin, Cat. abs9495), and seeded in 24-well plates. After 15 min, 500 μl of human lung cancer organoid medium A was added to each well. After 2 days of culturing, the medium was replaced.

For lentiviral transduction, organoids were dissociated into single cells and seeded in 24-well plates. Cells were then transduced with lentiviral particles carrying the human-TIMELESS-sgRNALGE-4 construct in the presence of 5 μg/ml polybrene to enhance infection efficiency. After 24 h, the cells were collected by centrifugation at 300 ×*g* for 5 min, resuspended in Matrigel, and replated. Following a recovery period for organoid proliferation, selection was initiated with 2 μg/ml puromycin for 5 days. The resulting puromycin-resistant organoids were then treated with 5 μmol/l erastin for 5 days, imaged using an Invitrogen EVOS M7000 inverted microscope, and the viability was quantified using ImageJ software (version 1.54p).

### Organoid viability assays

Control and experimental group organoids were seeded in Matrigel-coated, black-bottom 96-well plates at 100 organoids/well. Following a 5-day treatment with 5 μmol/l erastin, organoid viability was assessed using CellTiter-Glo 3D Cell Viability Assay (Promega, Cat. G9681). Luminescence was measured on a microplate reader (TECAN, Infinite 200 PRO). The luminescence values were normalized to the control group.

### Lewis lung carcinoma mouse model and therapeutic protocols

*Timeless*-knockdown (*shTimeless*) LLC1 cells were generated by transduction with *Timeless*-knockdown shRNA lentivirus (Tsingke; sequence is provided in Table S4). LLC1 cells transduced with control shRNA (*shCtrl*) or *shTimeless* were orthotopically injected into the left thoracic cavity of C57BL/6J mice via intercostal approach using a 30 G needle (5 × 10^5^ LLC1 cells in 50 μl of Matrigel), as previously described [41]. Ten days later, tumor formation was monitored by an In Vivo Imaging System (PerkinElmer, IVIS Spectrum). The animal study was reviewed and approved by the Ethics Committee of Air Force Medical University (No. 20230930).

For combination therapy, mice injected with *shCtrl* and *shTimeless* LLC1 cells were randomized into 4 groups: (a) DMSO plus isotype IgG2α (BioXcell, Cat. BE0089, 200 μg, injected intraperitoneally on days 10, 13, 16, 19, 22, 25, and 28); (b) erastin (30 mg/kg, injected intraperitoneally daily) plus isotype IgG2α; (c) DMSO plus anti-PD-1 antibody (BioXcell, Cat. BE0146, 200 μg, injected intraperitoneally on days 10, 13, 16, 19, 22, 25, and 28); and (d) erastin plus anti-PD-1 antibody. Tumor progression was monitored every 5 days using an In Vivo Imaging System. For ferroptosis inhibition studies, ferrostatin-1 was administered at 10 mg/kg every third day.

### Flow cytometry analysis

For flow cytometry analysis after in vivo experiments, the steps were briefly outlined below [42]. Mouse lung tumors were rapidly excised and mechanically dissociated in serum-free RPMI 1640 medium. Tissues were digested in serum-free RPMI 1640 supplemented with 1 mg/ml DNase I (Aladdin, Cat. D128589) and 1 mg/ml collagenase I (Sigma, Cat. SCR103) at 37 °C on a shaker for 10 to 30 min to dissociate into cell suspensions. Single-cell suspensions were passed through 70-μm cell strainers, and erythrocytes were lysed using red blood cell lysis buffer (BD, Cat. 349202) for 10 min at 4 °C. Cells were washed in PBS containing 2% FBS and subsequently incubated with TruStain FcX (BioLegend, Cat. 101319) for 10 min at 4 °C to prevent nonspecific antibody binding. Following Fc receptor blockade, cells were stained with fluorochrome-conjugated antibodies for 30 min at 4 °C. The antibodies used for flow cytometry are listed in Table S7. Samples were analyzed on an ACEA NovoCyte Flow Cytometer (Agilent, NovoCyte 3000RYB).

### Molecular docking analysis

Protein–protein docking between TIMELESS and CNOT3 was performed using the GRAMM Docking Web Server (https://gramm.compbio.ku.edu/). Three-dimensional structures of TIMELESS and CNOT3 were retrieved from the Protein Data Bank. Docking employed the standard Fast Fourier Transform algorithm to identify the lowest-energy binding conformation. Results were visualized using PyMOL software (version 3.0).

### Immunohistochemistry and hematoxylin–eosin staining

For organoid immunohistochemistry (IHC) staining: LUAD organoids (>200 μm diameter) were fixed in 4% PFA for 1 h, embedded in melted agarose, and processed through gradient ethanol dehydration and paraffin embedding. For tissue IHC staining: fresh human LUAD specimens, adjacent normal tissues, lung tumors, and xenografted tumors were fixed in 4% PFA overnight, then paraffin-embedded and sectioned at 3 to 4 μm using a rotary microtome.

For hematoxylin–eosin (H&E) staining, tissue sections were dewaxed, stained with hematoxylin (nuclear) and eosin (cytoplasmic), dehydrated, and mounted. For IHC staining, the tissue sections were dewaxed with xylene, antigen retrieval was performed, and the sections were incubated with the primary antibodies overnight at 4 °C, followed by secondary antibody incubation and staining with DAB reagent (ZSGB-BIO, Cat. ZLI-9017). Nuclei were counterstained with hematoxylin, followed by dehydration and sealing. The sections were scanned using a digital slide scanning system (WINMEDIC).

### Multiplex immunofluorescence

Mouse orthotopic lung tumor tissue sections and human LUAD TMA were analyzed by mIF using a total of 8 antibodies, as listed in Table S7. Briefly, the sections were dewaxed and hydrated. Endogenous horseradish peroxidase (HRP) was inhibited with 3% H_2_O_2_. After blocking for 30 min at room temperature, the slides were incubated with primary antibody for 1 h at room temperature or overnight at 4 °C, followed by incubation with HRP-labeled secondary antibody at room temperature for 10 min. Tyramide Signal Amplification (TSA) was then performed using tyramide-conjugated fluorophores for 10 min at 24 °C for signal amplification. This staining cycle was repeated for each antibody according to the above process. The TSA opal fluorophores used for mouse tumor sections were TG440N, TG570N, TG650N, and TG690N. For the LUAD TMAs, the corresponding tyramine signal amplification fluorescent dyes used were iF647, iF440, iF488, iF546, iF594, and iF750. After the last dye reaction, the nuclei were stained with the nuclear dye TG470SN before coverslip mounting. Image acquisition and positive cell analysis were performed on the sections using a TissueFAXS Spectra system (TissueGnostics).

### Tumorigenesis assay

For xenograft experiments, 6- to 8-week-old BALB/c nude mice were randomly divided into 3 groups (*n* = 5 per group). Each group received a subcutaneous injection of 1 × 10^6^ stably transfected H1975 cells corresponding to the nontargeting control, *TIMELESS*-knockout alone, or *TIMELESS*-knockout plus *TF*-knockdown, respectively. After 4 weeks, the mice were sacrificed, and the subcutaneous tumor tissues were removed, weighed, and photographed.

### Statistical analysis

All quantification results were presented as mean ± standard error of the mean obtained from at least 3 independent experiments unless otherwise indicated. Two-tailed Student’s *t* test was used for comparisons between 2 groups, while 2-way analysis of variance (ANOVA) was used for comparisons among 3 or more groups. The chi-square test or Fisher’s exact test was used to analyze correlations between TIMELESS expression and clinical characteristics. Survival curves were generated by the Kaplan–Meier method. Organoid size and immunoblot gray values were quantified using ImageJ software. Statistical analyses were performed with GraphPad Prism (version 10.1.2). *P* < 0.05 was considered statistically significant.

## Results

### Dysregulation of RBP expression affects LUAD patient survival

**Fig. 1.**
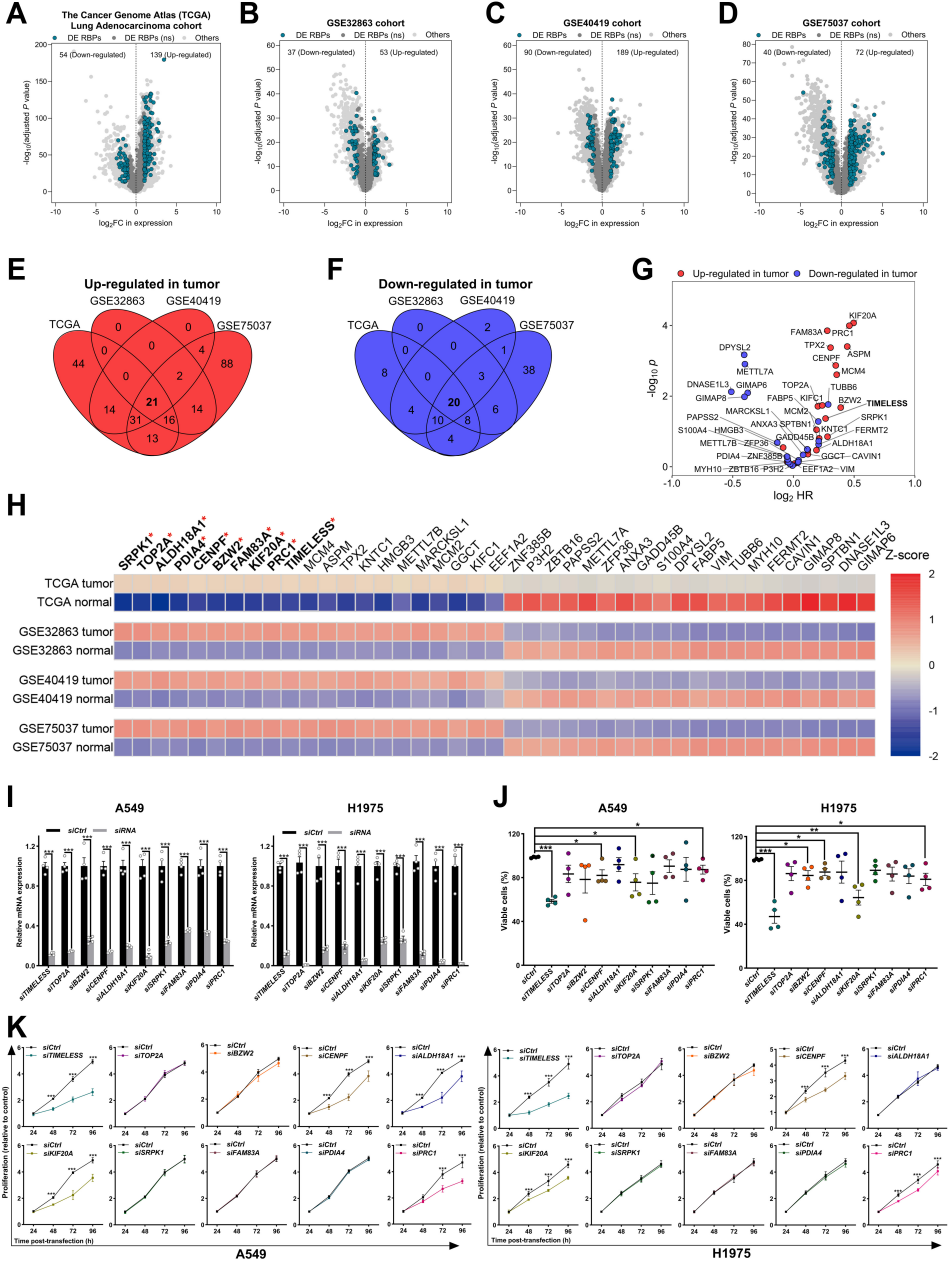
Dysregulated RNA-binding proteins in LUAD: from multicohort screening to functional validation. (A to D) Volcano plots showing the DE RBPs in the following 4 LUAD cohorts: The Cancer Genome Atlas Lung Adenocarcinoma (TCGA-LUAD) dataset (*n* = 568; A), GSE32863 (*n* = 116; B), GSE40419 (*n* = 164; C), and GSE75037 (*n* = 166; D). The significantly DE RBPs (*P*_adj_ < 0.05, |log_2_FC| > 1) are shown in blue, and the nonsignificantly DE RBPs (*P*_adj_ > 0.05, |log_2_FC| > 1) are shown in dark gray. The other non-RBP genes are shown in light gray. (E and F) Venn diagram showing the intersection of up-regulated (E) and down-regulated (F) DE RBPs across TCGA-LUAD and the 3 GEO datasets. (G) The volcano plot displayed the hazard ratios for patients stratified by the median expression level of RBP genes in the TCGA-LUAD cohort (red: up-regulated RBPs in tumors; blue: down-regulated RBPs in tumors). (H) Heatmap of significantly dysregulated RBPs identified in (E) and (F). The top 10 highest-expressed RBPs in tumors are highlighted (ranked by average tumor *z* score). (I) Expression levels of the top 10 LUAD-up-regulated RBPs in LUAD cell lines (A549 and H1975) with or without siRNA-mediated knockdown (*n* = 4 each group). (J) Calcein-AM/PI staining showed the percentage of viable cells in the negative control group and following the knockdown of the RBP gene (*n* = 4 each group). (K) CCK-8 assay of cell viability in RBP gene knockdown and control cells at the indicated time points. The statistical analysis was performed using a 2-tailed Student’s *t* test (I and J) or 2-way ANOVA (K). *P < 0.05, **P < 0.01, ***P < 0.001, data without statistically significant differences are not labeled (J and K). Abbreviations: ALDH18A1, aldehyde dehydrogenase 18 family member A1; BZW2, basic leucine zipper and W2 domains 2; CCK-8, cell counting kit-8; CENPF, centromere protein F; DE, differentially expressed; FAM83A, family with sequence similarity 83 member A; FC, fold change; FDR, false discovery rate; GEO, Gene Expression Omnibus; IF, immunofluorescence; IHC, immunohistochemistry; KIF20A, kinesin family member 20A; LUAD, lung adenocarcinoma; OS, overall survival; PDIA4, protein disulfide isomerase family A member 4; PRC1, protein regulator of cytokinesis 1; RBP, RNA-binding protein; SRPK1, SRSF protein kinase 1; TCGA, The Cancer Genome Atlas; TMA, tissue microarray; TOP2A, DNA topoisomerase II alpha.

Gene expression dysregulation represents a fundamental characteristic of cancer [43]. RBPs contribute to tumor progression by recognizing specific RNA-binding domains and post-transcriptionally modulating target gene function [44]. Analysis of the TCGA-LUAD dataset identified 139 significantly up-regulated and 54 down-regulated RBP genes in LUAD. Moreover, this imbalance toward up-regulation of RBP genes was consistently observed in the 3 independent GEO cohorts (Fig. 1A to D). Intersection analysis across datasets identified 21 up-regulated and 20 down-regulated RBPs with concordant expression patterns (Fig. 1E and F). Cox proportional hazards regression analysis revealed that up-regulated expression of RBPs was significantly associated with poorer patient survival (Fig. 1G and Fig. S1A). Based on *z*-score rankings, the top 10 significantly up-regulated RBPs in tumors, including both canonical and noncanonical types, were selected for further validation (Fig. 1H). Systematic evaluation of RBP expression in an independent GEO dataset (GSE283245) confirmed significant overexpression of 6 out of the 10 candidate RBPs in LUAD tissues compared with paired normal tissues (Fig. S1B). scRNA-seq analysis of 41 RBPs revealed expression trends consistent with the bulk RNA-seq datasets (Fig. S1C). Notably, elevated expression of most RBPs correlated with reduced overall survival in TCGA-LUAD (Fig. S1D). To define the biological roles of the top 10 up-regulated RBP genes, siRNA-mediated knockdown was performed in LUAD cell lines (A549 and H1975), and knockdown efficiency was confirmed by RT-qPCR (Fig. 1I). Silencing *TIMELESS*, *CENPF*, *KIF20A*, and *PRC1* significantly reduced viable cell counts (Fig. 1J) and diminished cell viability (Fig. 1K). The results indicated several dysregulated RBPs in LUAD that may serve as candidate biomarkers with prognostic potential.

### TIMELESS promotes LUAD tumor growth by suppressing ferroptosis and is associated with poor prognosis

**Fig. 2.**
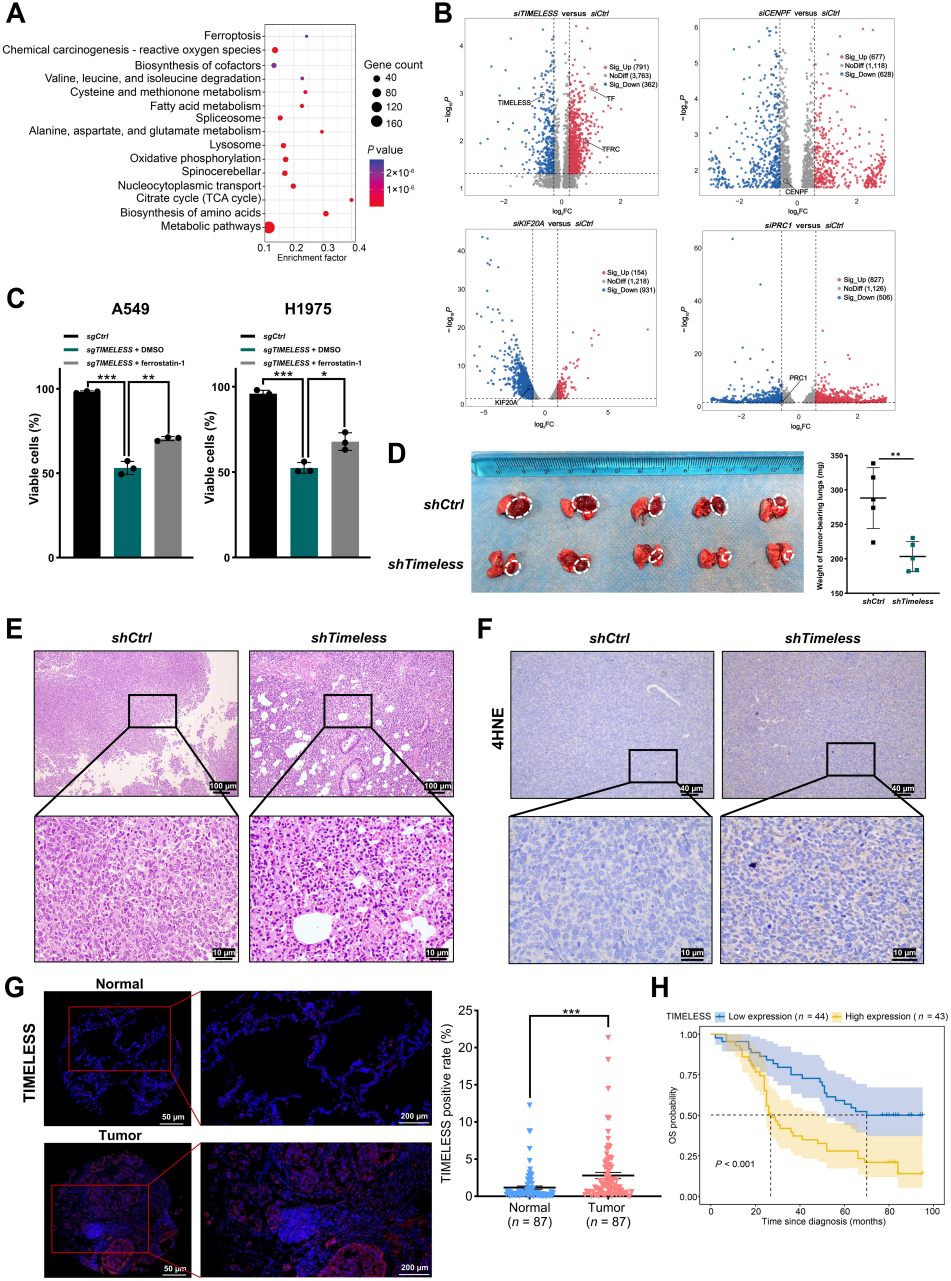
RBP TIMELESS promotes ferroptosis resistance and accelerates tumor progression in LUAD. (A) KEGG enrichment analysis of the up-regulated DEGs after *TIMELESS* knockdown. (B) Volcano plots of DEGs following RBPs (*TIMELESS, CENPF, KIF20A*, and *PRC1*) knockdown; the cutoffs for differential expression were set at |log_2_FC| > 1 and *P* < 0.05. Genes were categorized as follows: Sig_Up (significantly up-regulated), Sig_Down (significantly down-regulated), and NoDiff (nonsignificant). (C) Quantitative analysis of cell death rescued by ferrostatin-1 treatment, assessed by costaining with Calcein-AM/PI. (D) Images of lung tumors (white-dashed circle) excised at day 30 post-injection from LLC1 orthotopic models established with control (*shCtrl*) or *Timeless*-knockdown (*shTimeless*) LLC1 cells. The corresponding quantitative analysis of the weight of tumor-bearing lungs is shown on the right (*n* = 5 per group). (E) Representative H&E staining of LLC1-induced lung tumor tissues from the control (*shCtrl*) and *Timeless* knockdown (*shTimeless*) group. (F) Representative IHC staining images of the ferroptosis marker 4HNE in LLC1-induced lung tumor tissues from *shCtrl* and *shTimeless* groups. (G) Representative IF staining and quantification of TIMELESS expression in LUAD and paired normal adjacent tissues from cohort 1 patients (LUAD TMA, *n* = 87). (H) Kaplan–Meier analysis showing the association between TIMELESS expression with OS in clinical LUAD patient cohort 1 (*n* = 87). Patients were dichotomized into high and low TIMELESS expression groups based on IHC staining scores, using the median value as the cutoff. The statistical analysis was performed using a 2-tailed Student’s *t* test (C, D, and G). The survival analysis of LUAD patients was performed by the Kaplan–Meier method (H). *P < 0.05, **P < 0.01, ***P < 0.001. Abbreviations: 4HNE, 4-hydroxynonenal; DEGs, differentially expressed genes; H&E, hematoxylin and eosin; IHC, immunohistochemistry; KEGG, Kyoto Encyclopedia of Genes and Genomes; LLC, Lewis lung carcinoma; *shCtrl*, small hairpin RNA for the negative control; *shTimeless*, small hairpin RNA for *Timeless*; siRNA, small interfering RNA.

To dissect the mechanistic roles of the 4 selected RBPs (TIMELESS, CENPF, KIF20A, and PRC1) in LUAD, we conducted RNA-seq on corresponding knockdown cells and analyzed differentially expressed genes. GO and Reactome pathway analyses revealed that *TIMELESS* knockdown significantly enriched pathways related to RNA binding and metabolism. Among the 4 RBP knockdowns, only *TIMELESS* knockdown led to significant enrichment of the ferroptosis pathway in KEGG analysis, and induced pronounced alterations in the expression of ferroptosis-related genes (Fig. S2A and B and Fig. 2A and B). Based on the transcriptomic evidence, ferroptosis may contribute to the observed cell death. Consistently, cell death in *TIMELESS* knockdown cells was significantly rescued by the ferroptosis inhibitor ferrostatin-1 treatment (Fig. 2C and Fig. S2C). The physiological relevance of this functional interplay between TIMELESS and ferroptosis was validated in orthotopic models. *Timeless*-deficient tumors showed markedly reduced growth and nodule area (Fig. 2D and E), while exhibiting decreased Timeless and Ki67 expression alongside elevated levels of the ferroptosis biomarker 4HNE (Fig. 2F and Fig. S2D to F).

To determine the clinical significance of this oncogenic regulator, we analyzed TIMELESS expression in multiple independent datasets. Integrated assessment of IF, IHC, TCGA-LUAD, and scRNA-seq data identified TIMELESS as significantly up-regulated in tumor tissues compared with normal tissues (Fig. 2G and Fig. S2G to I). Systematic evaluation of TIMELESS expression patterns in LUAD TMAs revealed that high TIMELESS expression was significantly associated with aggressive clinicopathological parameters, including larger tumor size (>3 cm), later AJCC stage (III), and higher T stage (T3 to T4) (Table 1). Additionally, high TIMELESS expression was associated with poorer overall survival (Fig. 2H). Collectively, these findings suggested that TIMELESS may function as an important oncogenic regulator in LUAD and implied that the suppression of ferroptosis could be a contributing mechanism to its tumor-promoting function.

### TIMELESS knockout promotes ferroptosis in LUAD cells and patient-derived organoids

**Fig. 3.**
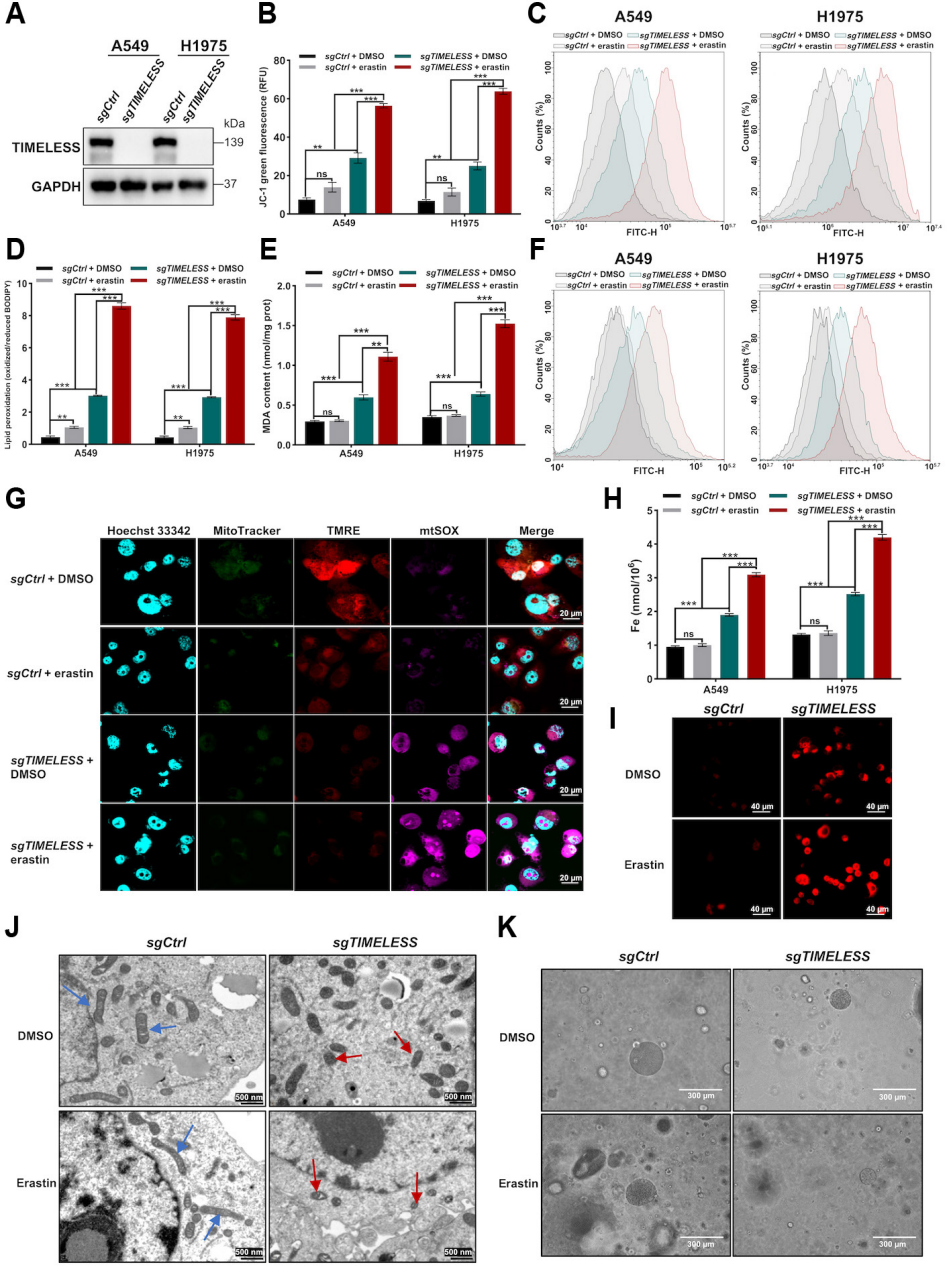
TIMELESS deficiency enhances ferroptosis susceptibility in LUAD cells and organoids. (A) Western blotting analysis of TIMELESS protein expression in A549 and H1975 cells transduced with control (*sgCtrl*) or *TIMELESS* targeting (*sgTIMELESS*) sgRNAs. (B) MMP was assessed by JC-1 flow cytometry in *sgCtrl* and *sgTIMELESS* cells following a 48-h treatment with either DMSO or erastin (3 or 5 μmol/l for H1975 or A549 cells, respectively; *n* = 3 per group). (C) Intracellular total ROS were assessed by DCFH-DA fluorescence in A549 and H1975 cells after 48-h treatments with DMSO or erastin. (D) Lipid peroxidation was monitored using BODIPY 581/591 C11 staining. Experimental groups were as follows: *sgCtrl* + DMSO, *sgCtrl* + erastin, *sgTIMELESS* + DMSO, and *sgTIMELESS* + erastin. (E) MDA content was detected in *sgCtrl* and *sgTIMELESS* cells following DMSO or erastin treatment. (F) Lipid peroxides were assessed using the Liperfluo probe and flow cytometry in *sgCtrl* and *sgTIMELESS* cells treated with DMSO or erastin for 48 h. (G) Multiplexed imaging of nuclei (Hoechst 33342, blue), mitochondrial mass (MitoTracker Green FM, green), MMP (TMRE, red), and mitochondrial superoxide (mtSOX deep Red, purple) in *sgCtrl* and *sgTIMELESS* H1975 cells treated with either DMSO or erastin. (H) Total iron content was quantified using a colorimetric assay in the indicated groups. (I) Labile Fe^2+^ levels were assessed by FerroOrange staining in H1975 cells from the following groups: *sgCtrl* + DMSO, *sgCtrl* + erastin, *sgTIMELESS* + DMSO, and *sgTIMELESS* + erastin. (J) TEM images of mitochondria in *sgCtrl* and *sgTIMELESS* H1975 cells treated with either DMSO or erastin. Blue arrows indicate mitochondria with obvious cristae, while red arrows indicate shrunken mitochondria. (K) Representative micrographs of *sgCtrl* and *sgTIMELESS* LUAD organoids treated with DMSO or erastin. The statistical analysis was performed using a 2-tailed Student’s *t* test (B, D, E, and H). *P < 0.05, **P < 0.01, ***P < 0.001, ns: not significant. Abbreviations: DCFH-DA, 2′,7′-dichlorodihydrofluorescein diacetate; DMSO, dimethyl sulfoxide; MDA, malondialdehyde; MMP, mitochondrial membrane potential; mtSOX, mitochondrial superoxide; ROS, reactive oxygen species; TEM, transmission electron microscopy; TMRE, tetramethylrhodamine ethyl ester.

To delineate the ferroptosis-modulatory role of TIMELESS, we validated silencing efficacy via Western blotting and RT-qPCR analyses (Fig. 3A and Fig. S3A). In *TIMELESS* knockout cells, erastin exhibited a reduced IC_50_, and *TIMELESS* knockout cells exhibited reduced post-treatment viability (Fig. S3B to C). To assess the functional impact on mitochondria, changes in MMP were measured using JC-1 staining. Erastin treatment potentiated the loss of MMP in *TIMELESS* knockout cells (Fig. 3B and Fig. S3D). Metabolic activity is accompanied by the production of ROS, which can cause lipid peroxidation of the cell membrane [45]. Erastin treatment augmented the accumulation of ROS and lipid peroxidation in *TIMELESS* knockout A549 and H1975 cells (Fig. 3C and D and Fig. S3E). MDA, a lipid peroxidation product, was significantly elevated in erastin-treated *TIMELESS* knockout cells (Fig. 3E). Liperfluo is specifically oxidized by lipid peroxides associated with intracellular ferroptosis and emits intense fluorescence [46]. *TIMELESS* knockout accelerated the ability of erastin to produce lipid peroxides, as measured with the Liperfluo probe (Fig. 3F). Compared with control cells, mitochondrial superoxide production was increased in *TIMELESS* knockout cells after erastin addition, as assessed by mtSOX staining (Fig. 3G). Total iron and ferrous iron (Fe^2+^) are important indicators to determine the occurrence of ferroptosis [47]. *TIMELESS* knockout manifested markedly higher levels of total iron and Fe^2+^ compared to control cells in the presence of erastin (Fig. 3H and I). Mitochondrial shrinkage was observed in *TIMELESS* knockout H1975 cells by TEM, and this shrinkage was exacerbated by erastin treatment (Fig. 3J). Consistent with the effects of erastin, treatment with the ferroptosis inducer RSL3 also enhanced the sensitivity of *TIMELESS* knockout cells to ferroptosis (Fig. S4). Compared with RSL3 treatment, quantitative analyses indicated that erastin treatment induced more pronounced ferroptosis manifestations in LUAD cells (Fig. S5A to C). Thus, erastin was selected for subsequent experiments.

To evaluate the effects of erastin and *TIMELESS* knockout, LUAD patient-derived organoids (PDOs) were established as a preclinical model. IHC analysis confirmed that LUAD PDOs retained the histological features of the originating tumors (Fig. S5D). The efficiency of *TIMELESS* knockout in CRISPR-Cas9-edited organoids was verified by IHC, with a concomitant increase in 4HNE expression (Fig. S5E and F). Consistent with the cellular observations, erastin treatment suppressed organoid size and viability in *TIMELESS* knockout PDOs (Fig. 3K and Fig. S5G and H). Together, these results demonstrated that *TIMELESS* knockout sensitizes LUAD cell lines and PDOs to ferroptosis inducers.

### TIMELESS binds to and post-transcriptionally regulates TF mRNA in LUAD cells

**Fig. 4.**
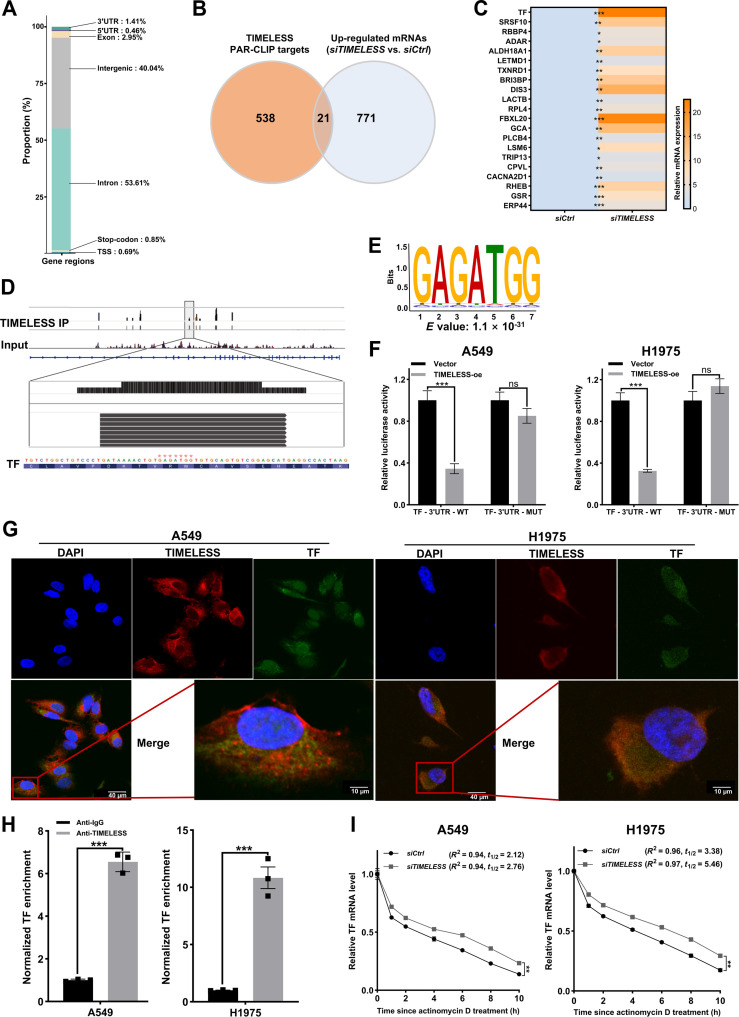
TIMELESS inhibits ferroptosis through TF down-regulation in LUAD. (A) Distribution of TIMELESS PAR-CLIP binding sites across genomic features. (B) Overlap between TIMELESS PAR-CLIP targets and up-regulated mRNAs upon its knockdown in H1975 cells. (C) RT-qPCR validation of the 21 overlapping genes (B) in *siCtrl* and *siTIMELESS* H1975 cells. (D) TIMELESS PAR-CLIP peaks in the TF transcript in H1975 cells. Mapping of the TIMELESS-binding site to the GAGATGG motif located in TF 3′UTR. TIMELESS IP: immunoprecipitated with an anti-TIMELESS antibody. Input: whole cell lysate control. (E) The de novo discovered RNA-binding motif for TIMELESS was identified through PAR-CLIP-seq and its significance was analyzed using the MEME suite. (F) Luciferase activity of wild-type (WT) or mutant (MUT) TF 3′UTR reporter plasmid was measured in A549 and H1975 cells cotransfected with a TIMELESS-overexpression plasmid (TIMELESS-oe) or an empty vector control (Vector). (G) Localization of TIMELESS protein (red) and TF mRNA (green) in A549 and H1975 cells, as detected by IF and RNA-FISH assays, respectively. (H) RIP verified the binding of TF mRNA to TIMELESS in A549 and H1975 cells. IgG antibody served as the negative control. (I) The decay of TF mRNA was measured after actinomycin D (2.5 μmol/l) treatment in *siCtrl* and *siTIMELESS* A549 and H1975 cells. The mRNA half-life (*t*_1/2_) was derived from the best-fit curve (*R*^2^ values shown). The statistical analysis was performed using a 2-tailed Student’s *t* test (C, F, and H) and one-phase decay modeling for half-life calculation (I). *P < 0.05, **P < 0.01, ***P < 0.001, ns: not significant. Abbreviations: FISH, fluorescence in situ hybridization; GO, Gene Ontology; IF, immunofluorescence; IP, immunoprecipitation; PAR-CLIP, photoactivatable ribonucleoside-enhanced crosslinking and immunoprecipitation; RT-qPCR, reverse transcription quantitative real-time PCR; RIP, RNA immunoprecipitation; TF, transferrin; UTR, untranslated region.

To identify RNAs directly bound by TIMELESS, PAR-CLIP analyses were performed in H1975 cells. Binding peaks were annotated across genomic regions, including introns, intergenic regions, exons, 3′UTR, 5′UTR, stop-codon, and transcription start site (TSS) (Fig. 4A). By integrating PAR-CLIP-seq and RNA-seq data from *TIMELESS* knockdown cells, we identified transcripts that are both direct targets of TIMELESS and under its transcriptional repression. In total, we discovered 21 genes that were directly bound by TIMELESS at the mRNA level and significantly up-regulated upon *TIMELESS* knockdown (Fig. 4B). RT-qPCR validation confirmed the consistent up-regulation patterns of these targets (Fig. 4C). Moreover, pathway analysis revealed that the identified TIMELESS target genes were significantly enriched in ferroptosis (Fig. S6A). By focusing on ferroptosis regulators that aligned with phenotypic observations, TF was identified as a key candidate regulator of ferroptosis (Fig. 4D). PAR-CLIP analysis of TIMELESS revealed a conserved heptanucleotide motif (GAGATGG) in the TF 3′UTR (Fig. 4E). Dual-luciferase assays confirmed TIMELESS-mediated post-transcriptional regulation through TF 3′UTR binding (Fig. 4F), and no transcriptional modulation was observed (Fig. S6B). Subsequently, cytoplasmic and nuclear proteins were extracted from human normal bronchial epithelial cells and LUAD cells. Subcellular fractionation showed the presence of TIMELESS along with iron metabolism-related molecules: TF, transferrin receptor (TFRC), 6-transmembrane epithelial antigen of prostate 3 (STEAP3), and divalent metal transporter 1 (DMT1) in both cellular compartments (Fig. S6C). To determine whether TIMELESS interacts with *TF* mRNA in cells, an IF–fluorescence in situ hybridization (FISH) assay was performed, which showed a distinct colocalization of TIMELESS protein with *TF* mRNA (Fig. 4G). RIP assays further confirmed this specific RNA–protein interaction, as evidenced by significant enrichment of *TF* mRNA in TIMELESS immunoprecipitants compared with control IgG (Fig. 4H). To investigate the potential role of TIMELESS in regulating *TF* mRNA stability, actinomycin D was used to block transcription, and *TF* mRNA levels were subsequently monitored in A549 and H1975 cells. Compared with control cells, *TIMELESS* knockdown cells exhibited enhanced stability and prolonged half-life of *TF* mRNA (Fig. 4I and Fig. S6D). Moreover, *TIMELESS* deficiency led to increased mRNA and protein levels of TF, TFRC, STEAP3, and DMT1 (Fig. S6E and F). These data suggested that TIMELESS is a critical regulator of LUAD proliferation, potentially through targeting TF, a pivotal factor in cellular iron metabolism.

### TF is essential for ferroptosis and tumor suppression induced by TIMELESS knockdown

**Fig. 5.**
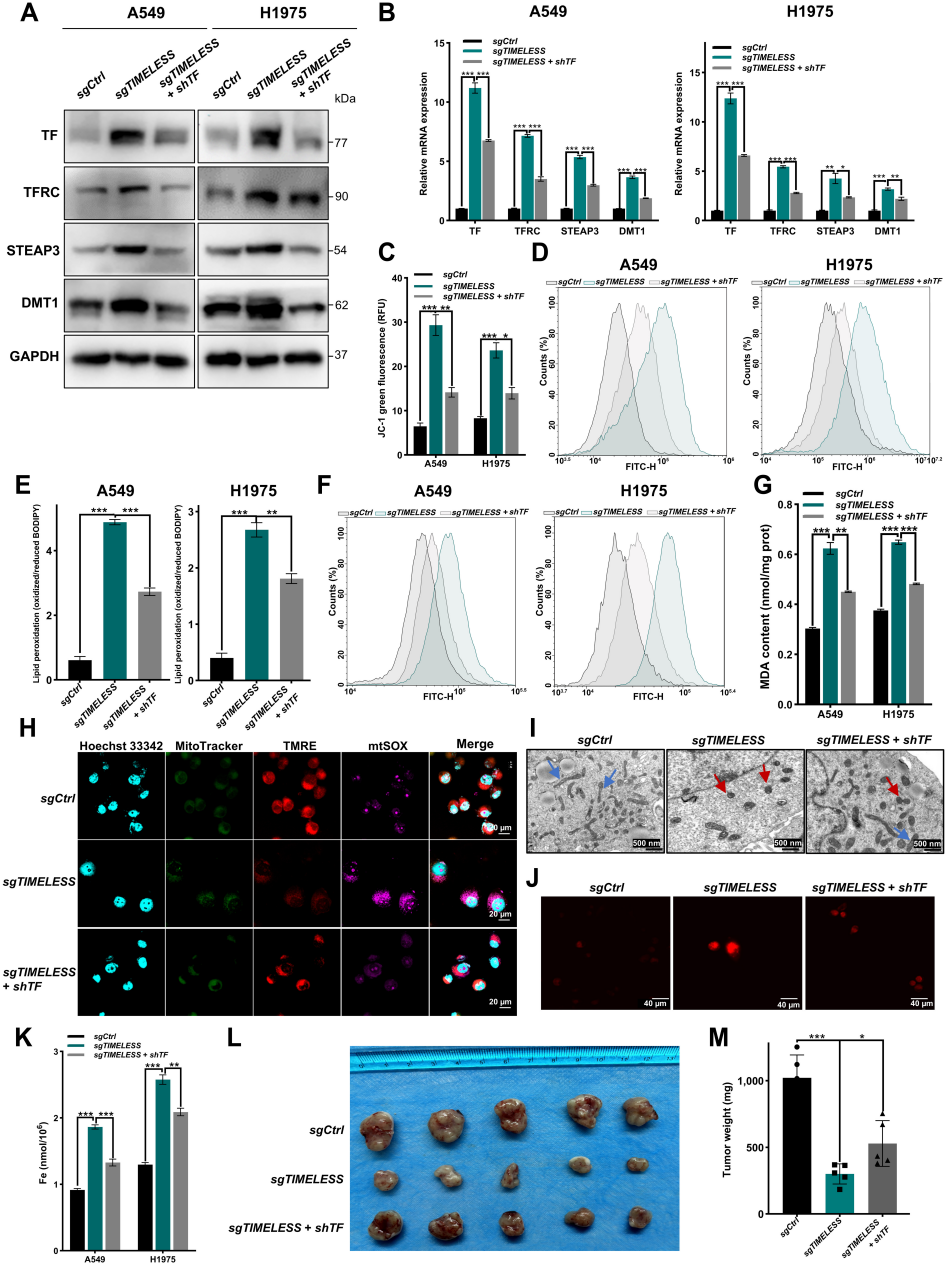
TF silencing partially alleviates ferroptosis and restores tumor growth in TIMELESS knockout models. (A and B) Western blotting (A) and RT-qPCR (B) were performed in control (*sgCtrl*), *TIMELESS*-knockout (*sgTIMELESS*), and *TIMELESS*-knockout followed by *TF*-knockdown (*sgTIMELESS + shTF*) A549 and H1975 cells. (C) Flow cytometric analysis of MMP using JC-1 in *sgCtrl*, *sgTIMELESS*, and *sgTIMELESS* + *shTF* A549 and H1975 cells. (D) ROS production in *sgCtrl*, *sgTIMELESS*, and *sgTIMELESS* + *shTF* cells. (E) Lipid peroxidation accumulation was assessed in *sgCtrl*, *sgTIMELESS*, and *sgTIMELESS* + *shTF* A549 and H1975 cells using BODIPY 581/591 C11 staining. (F) Lipid peroxides were monitored with Liperfluo fluorescence probe in the indicated groups. (G) MDA levels were measured in A549 and H1975 cells from the following groups: *sgCtrl*, *sgTIMELESS*, and *sgTIMELESS* + *shTF*. (H) Nuclei (Hoechst 33342, blue), mitochondrial mass (MitoTracker Green FM, green), MMP (TMRE, red), and mitochondrial superoxide (mtSOX deep Red, purple) were simultaneously stained in the indicated cell groups. (I) Representative TEM images of mitochondria in H1975 cells from the *sgCtrl*, *sgTIMELESS*, and *sgTIMELESS* + *shTF* groups. (J) Intracellular Fe^2+^ was detected using the FerroOrange probe in the indicated groups. (K) Total iron content was quantified in A549 and H1975 cells from the *sgCtrl*, *sgTIMELESS*, and *sgTIMELESS* + *shTF* groups. (L and M) *In vivo* tumor growth was shown by representative images (L) and tumor weight quantification (M) from xenograft models. The statistical analysis was performed using a 2-tailed Student’s *t* test (B, C, E, G, K, and M). *P < 0.05, **P < 0.01, ***P < 0.001. Abbreviations: MDA, malondialdehyde; MMP, mitochondrial membrane potential; mtSOX, mitochondrial superoxide; ROS, reactive oxygen species; TEM, transmission electron microscopy; TMRE, tetramethylrhodamine ethyl ester.

Iron uptake occurs primarily via the TF/TFRC transport axis [48]. To validate the clinical relevance of TF, the significant down-regulation of TF was confirmed in LUAD using paired clinical specimens (Fig. S7A). In addition, analysis of TMAs corroborated the reduced TF protein levels in LUAD tissue (Fig. S7B). Consistently, low TF expression was significantly associated with more advanced disease stage (AJCC stage III or T3 to T4) (Table 1). Analysis of LUAD TMAs and TCGA-LUAD revealed that elevated TF expression was significantly associated with improved overall survival (Fig. S7C and D). According to the GEO database, *TF* expression was inversely associated with *TIMELESS* expression levels (Fig. S7E). To validate the necessity of TF in TIMELESS-mediated ferroptosis, *TF*-specific shRNA was introduced into *TIMELESS* knockout cells. Western blotting and RT-qPCR analyses revealed down-regulation of iron metabolism-related genes after down-regulation of both *TIMELESS* and *TF* (Fig. 5A and B and Fig. S7F), and *TF* silencing reversed the ferroptotic phenotype induced by *TIMELESS* ablation, as evidenced by a significant increase in cell viability (Fig. S7G) and rescued the MMP (Fig. 5C and Fig. S7H). Moreover, knockdown of both *TIMELESS* and *TF* reversed multiple markers of oxidative damage, including total ROS, lipid peroxidation, lipid peroxides, and MDA levels (Fig. 5D to G and Fig. S7I), along with a phenotypic attenuation in mitochondrial superoxide (Fig. 5H). Silencing of both *TIMELESS* and *TF* ameliorated ultrastructural mitochondrial shrinkage (Fig. 5I) and restored intracellular iron homeostasis (Fig. 5J and K). Rescue experiments confirmed the ability of TF sufficiency to reverse TIMELESS-mediated ferroptosis resistance. In *TIMELESS* overexpressing LUAD cells, *TF* overexpression enhanced lipid peroxide generation, amplified lipid peroxidation, and augmented iron accumulation, effectively reversing ferroptosis execution (Fig. S8). Together, these results suggest that TIMELESS governs ferroptosis susceptibility through TF-dependent dysregulation of labile iron pool dynamics and lipid peroxidation cascades.

To define the functional impact of this regulatory mechanism, TIMELESS-TF interplay was assessed in vivo. Subcutaneous xenograft models revealed that *TF* knockdown attenuated tumor suppression mediated by *TIMELESS* ablation (Fig. 5L and M). Compared with the baseline established by *TIMELESS* knockdown, tumors with the double *TIMELESS* and *TF* knockdown exhibited reduced lipid peroxidation signatures and partial restoration of proliferative capacity as measured by Ki67 expression (Fig. S9A to C). Compared with the *Timeless* knockdown group, mice bearing *Timeless* and *Tf* double knockdown LLC1 cells (Fig. S9D and E) showed reversal tumor growth, manifested by increased pulmonary tumor burden and elevated Ki67 expression, concomitant with restored iron homeostasis and attenuated ferroptosis damage (Fig. S9F to I). Overall, these findings demonstrated that TF down-regulation reverses the pro-ferroptotic phenotype resulting from TIMELESS deficiency across tumor models, possibly establishing it as the core mechanism of this regulatory axis.

### TIMELESS recruits CNOT3 to degrade TF mRNA and inhibit ferroptosis

**Fig. 6.**
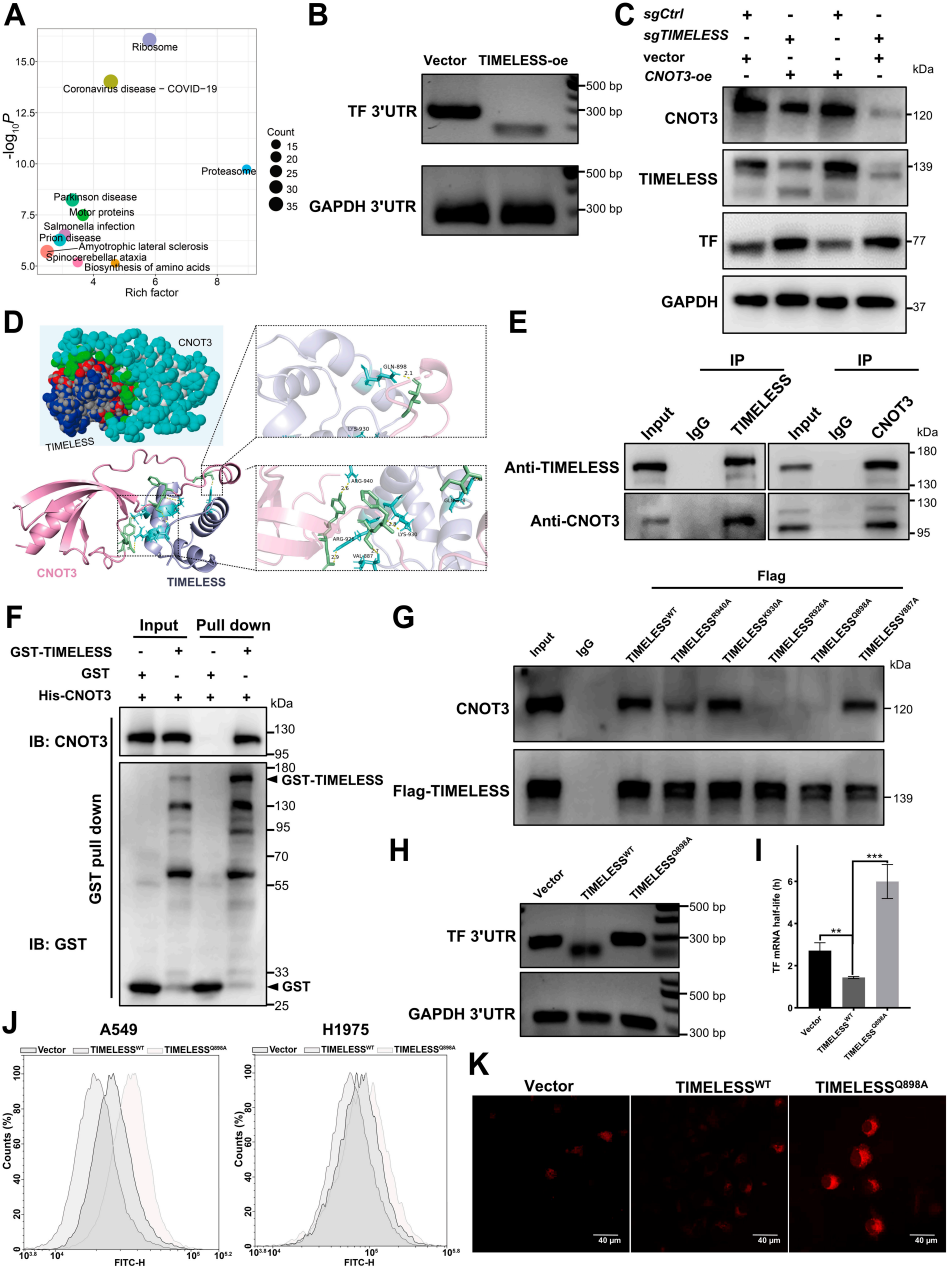
TIMELESS recruits CNOT3 to degrade TF mRNA, and their interaction mediates ferroptosis suppression. (A) KEGG pathway enrichment of TIMELESS-interacting proteins identified by IP-MS. (B) Poly(A) tail length of TF mRNA was assessed in H1975 cells overexpressing TIMELESS (TIMELESS-oe) using a quantitative PCR-based assay. GAPDH mRNA served as an internal control. (C) Representative Western blotting showing the protein levels of CNOT3, TIMELESS, and TF in H1975 cells, including control of *TIMELESS* knockout + control of *CNOT3* overexpression (*sgCtrl* + vector), *TIMELESS* knockout + *CNOT3* overexpression (*sgTIMELESS* + *CNOT3*-oe), control of *TIMELESS* knockout + *CNOT3* overexpression (*sgCtrl* + *CNOT3*-oe), and *TIMELESS* knockout + control of *CNOT3* overexpression (*sgTIMELESS* + vector). (D) Predicted structural model of the TIMELESS–CNOT3 complex obtained by molecular docking simulation. (E) Endogenous co-IP of TIMELESS and CNOT3 in H1975 cells. Cell lysates were immunoprecipitated with anti-TIMELESS, anti-CNOT3, or anti-IgG, followed by immunoblotting with the antibodies against TIMELESS and CNOT3. (F) GST pull-down assay of the direct interaction between TIMELESS and CNOT3. Purified His-CNOT3 was incubated with GST or GST-TIMELESS; proteins pulled down were analyzed by immunoblotting with the indicated antibodies. (G) Co-IP validation of the TIMELESS–CNOT3 interaction using flag-tagged wild-type TIMELESS (TIMELESS^WT^) and TIMELESS mutants. (H) The poly(A) tail length of TF mRNA was assessed in H1975 cells stably expressing either flag-tagged wild-type TIMELESS (TIMELESS^WT^) or the flag-tagged Q898A mutant (TIMELESS^Q898A^). (I) TF mRNA half-life was calculated from actinomycin D-based transcription inhibition assays in H1975 cells expressing an empty vector control (Vector), TIMELESS^WT^, or TIMELESS^Q898A^. (J) Lipid peroxides were assessed using the Liperfluo probe in H1975 cells expressing vector, TIMELESS^WT^, or TIMELESS^Q898A^. (K) Intracellular Fe^2+^ measurement by FerroOrange staining in the indicated H1975 cells. The statistical analysis was performed using a 2-tailed Student’s *t* test (I). **P < 0.01, ***P < 0.001. Abbreviations: CNOT3, Ccr4-Not transcription complex subunit 3; Co-IP, co-immunoprecipitation; GST, glutathione *S*-transferase; IP-MS, immunoprecipitation–mass spectrometry; KEGG, Kyoto Encyclopedia of Genes and Genomes; RNA-seq, RNA sequencing.

To elucidate the mechanism of TIMELESS-mediated TF degradation, multiomics data were leveraged to explore post-transcriptional regulation, focusing on the evolutionarily conserved Ccr4-Not complexes that link mRNA decay to translational control [49,50]. IP-MS analysis identified a specific TIMELESS–CNOT3 interaction, which was supported by KEGG and GO enrichment analyses of ribosome-associated processes and RNA binding functions (Fig. 6A and Fig. S10A and B). PAT analysis demonstrated TIMELESS-accelerated deadenylation of *TF* mRNA (Fig. 6B). Multiomics analysis indicated the coordinated expression of TIMELESS and CNOT3 at both the transcriptomic and proteomic levels (Fig. S10C and D). TCGA-LUAD analysis indicated high *CNOT3* expression and a significant positive correlation between *TIMELESS* and *CNOT3* in tumor tissues (Fig. S10E and F), revealing the clinical relevance of this coordination. Western blotting, RT-qPCR, and IF analyses were used to investigate the functional consequence of this relationship, which showed that *TIMELESS* knockdown attenuated the CNOT3-mediated repression of TF (Fig. 6C and Fig. S10G to J). Collectively, these data suggested that TIMELESS interacts with CNOT3 to regulate TF expression.

To define the structural basis of the TIMELESS–CNOT3 interaction, we employed molecular docking to predict a compatible interface between the 2 proteins (Fig. 6D). This prediction was biochemically validated by co-IP, confirming endogenous binding in H1975 cells, and by GST pull-down, which established a direct physical interaction in vitro (Fig. 6E and F). To elucidate the critical binding residues, co-IP analysis of TIMELESS point mutants (R940A, K930A, R926A, Q898A, and V887A) was conducted. The results showed that the R940A, R926A, and Q898A mutants reduced the intensity of the TIMELESS–CNOT3 interaction band compared to the WT, with the Q898A mutant causing the most pronounced reduction (Fig. 6G). Compared with TIMELESS^WT^, the TIMELESS^Q898A^ mutant reduced deadenylation efficiency in PAT assays (Fig. 6H), and this impairment of mRNA decay was further supported by its significant inhibition of actinomycin D-induced *TF* mRNA degradation (Fig. S11A) and prolonged *TF* mRNA half-life (Fig. 6I). Compared with the TIMELESS^WT^ controls, impairment of the TIMELESS–CNOT3 complex function via the TIMELESS^Q898A^ mutant elevated key ferroptosis markers, including lipid peroxidation, intracellular Fe^2+^, MDA, and total iron content (Fig. 6J and K and Fig. S11B to D). These findings implicated CNOT3 in the mechanism of TIMELESS-directed *TF* mRNA degradation, suggesting that it may serve a regulatory function.

### TIMELESS down-regulation potentiates the therapeutic efficacy of erastin combined with immune checkpoint blockade and reprograms the TME

**Fig. 7.**
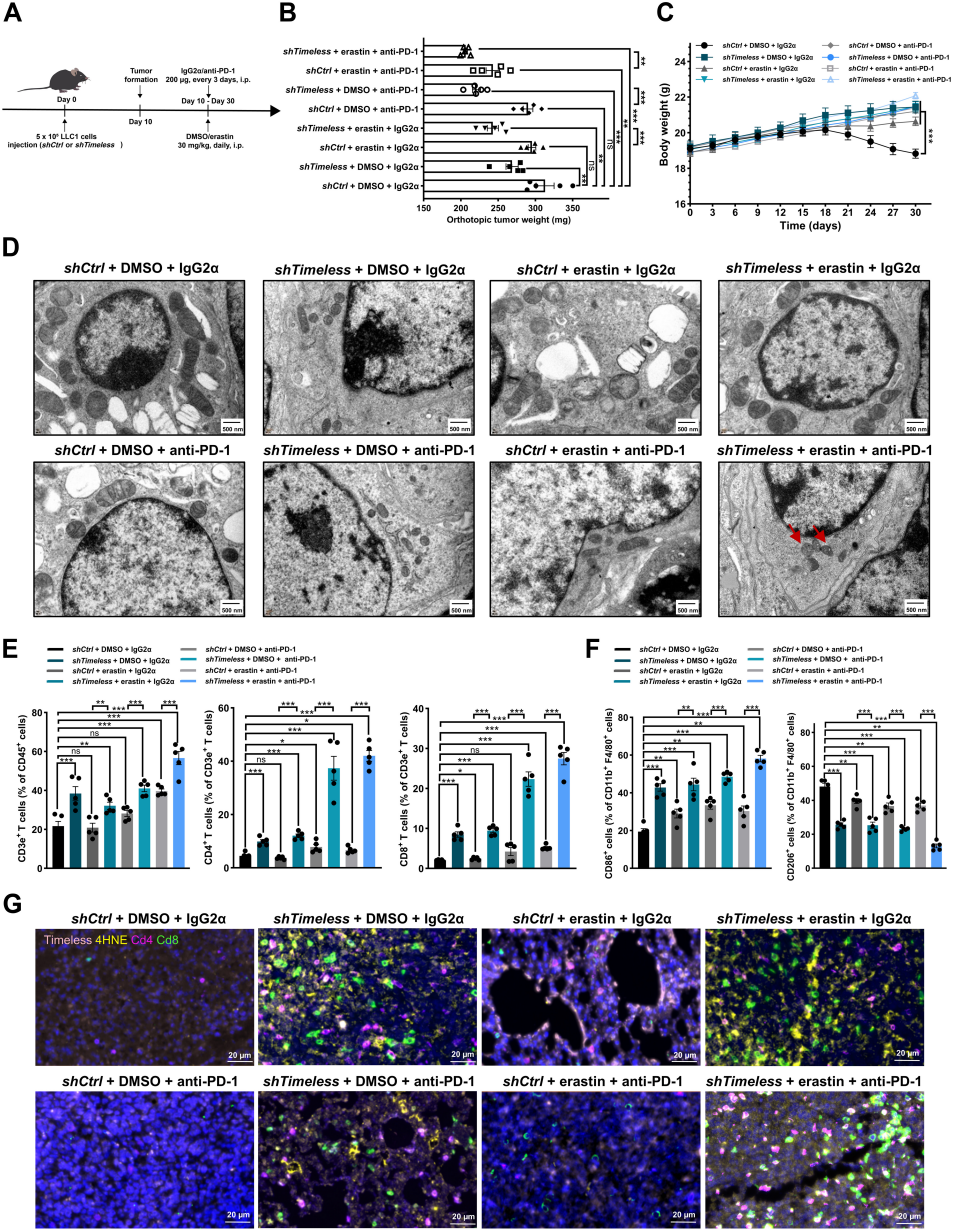
Timeless down-regulation potentiates erastin combined with anti-PD-1 therapy efficacy. (A) Experimental design for testing the combination of erastin and anti-PD-1 in an orthotopic LLC1 model. Ten days after orthotopic inoculation with 5 × 10^5^ *shCtrl* or *shTimeless* LLC1 cells, tumor-bearing mice were treated for 20 days with the following regimens: DMSO + IgG2α, erastin (30 mg/kg i.p. daily) + IgG2α, DMSO + anti-PD-1 (200 μg i.p. every 3 days), or erastin + anti-PD-1. (B) Weights of lung tumors after treatment in the orthotopic LLC1 model (*shCtrl* and *shTimeless*: DMSO + IgG2α, erastin + IgG2α, DMSO + anti-PD-1, and erastin + anti-PD-1). (C) Mice body weight was monitored every 3 days (*n* = 5). (D) Representative TEM images of mitochondria in lung tumors from orthotopic tumor-bearing mice transfected with *shCtrl* or *shTimeless* and treated with monotherapy or combination therapy. (E) Bar graphs illustrated the percentage of CD3e^+^ T cells among CD45^+^ cells, CD4^+^ T cells among CD3e^+^ T cells, and CD8^+^ T cells among CD3e^+^ T cells in lung orthotopic tumors. (F) CD86^+^ (M1-like) and CD206^+^ (M2-like) macrophage proportions among CD11b^+^F4/80^+^ cells in lung orthotopic tumors. (G) Representative mIF images of LLC1 tumor tissues from the *shCtrl* and *shTimeless* groups treated with the indicated regimens: DMSO + IgG2α, erastin + IgG2α, DMSO + anti-PD-1, and erastin + anti-PD-1. Staining shows Timeless (pink), 4HNE (yellow), Cd4 (purple), and Cd8 (green). Statistical analyses in (B), (E), and (F) were performed using a 2-tailed Student’s *t* test. Primary comparisons were made between each treatment group and the *shCtrl* + DMSO + IgG2α control. The significance is denoted above the bars. Further comparisons were conducted between the monotherapy and combination therapy groups, as well as between the *shTimeless* and *shCtrl* groups. *P < 0.05, **P < 0.01, ***P < 0.001, ns: not significant. Abbreviations: 4HNE, 4-hydroxyonenal; i.p., intraperitoneal; mIF, multiplex immunofluorescence; PD-1, programmed cell death protein 1; TEM, transmission electron microscopy.

To investigate the potential enhanced antitumor effect of combining ferroptosis induction with immunotherapy, an orthotopic lung cancer model was generated through intrapulmonary implantation of *Timeless* knockdown or control LLC1 cells, followed by treatment with erastin and anti-PD-1 antibody (Fig. 7A). Tumor volume measurements showed that the combination therapy produced enhanced suppression in *Timeless* knockdown tumors compared with the identically treated control tumors (Fig. 7B and Fig. S12A). Concurrent monitoring of physiological parameters showed that mice in the control group exhibited progressive weight loss, while all treatment groups maintained stable body weights throughout the treatment period (Fig. 7C). Survival analysis indicated extended survival in *Timeless* knockdown mice under combination therapy (Fig. S12B). Live imaging was performed 10 days after LLC1 cells implantation, and images were acquired every 5 days during the treatment. Tumor growth was significantly inhibited to a greater extent in the *Timeless* knockdown group treated with the combination therapy compared with the control group (Fig. S12C). IF analysis of Ki67 showed a decrease in proliferating cells within the *Timeless* knockdown tumors (Fig. S12D). The Fe^2+^, total iron, circulating TF levels, and mitochondrial changes were further analyzed in the orthotopic tumors derived from *Timeless* knockdown and control cells under different treatments. Compared with the control group, the *Timeless* knockdown tumor group exhibited significantly elevated levels of Fe^2+^, total iron, and circulating TF after combination treatment (Fig. S13A to C). TEM identified increased mitochondrial shrinkage in *Timeless* knockdown tumors after combination therapy (Fig. 7D). H&E staining of major organs (heart, liver, lung, and kidney) revealed intact morphology in all treatment groups. The lungs in the control group exhibited pathological alterations, including disrupted alveolar architecture, elevated nuclear-to-cytoplasmic ratios, and inflammatory infiltration, whereas the lungs of *Timeless* knockdown mice under combination therapy showed no lung injury, confirming the safety of erastin, anti-PD-1, and combined regimens in orthotopic models (Fig. S13D).

Numerous studies have reported that ferroptosis induction activates the immune system in response to tumor therapy and enhances the efficacy of immunotherapy [51,52]. Clinical evidence implicates TIMELESS in coordinating ferroptosis and immune evasion in LUAD. scRNA-seq analysis of LUAD patients receiving neoadjuvant PD-1 blockade with chemotherapy revealed that decreased TIMELESS expression in tumor cells was associated with enhanced immune infiltration, particularly of macrophages and T cells (Fig. S14A and B). Consistently, TCGA-LUAD analysis demonstrated inverse correlations between TIMELESS expression and ferroptosis scores, as well as the abundance of macrophages and CD4^+^ T cells (Fig. S14C). To investigate the role of TIMELESS in ferroptosis, pharmacological inhibition and induction were performed using ferrostatin-1 and erastin, respectively. Our data showed that in control groups, erastin treatment increased CD4^+^ and CD8^+^ T cell infiltration. This infiltration was further enhanced in the *Timeless* knockdown group. However, administration of ferrostatin-1 in the *Timeless* knockdown group reduced the expression of the ferroptosis marker 4HNE and concurrently attenuated T cell infiltration relative to the DMSO-treated *Timeless* knockdown group. In the *Timeless* knockdown group, ferrostatin-1 treatment also resulted in a significant increase in tumor weight compared with the DMSO-treated *Timeless* knockdown group (Fig. S15). To further examine the synergy between ferroptosis induction and remodeling of the tumor immune microenvironment, the infiltration of T cells and macrophages was evaluated in orthotopic mice treated with erastin combined with PD-1 blockade. Flow cytometry analysis showed that *Timeless* knockdown combined with erastin and anti-PD-1 increased the proportion of CD3e^+^ T cells, CD4^+^ T cells, CD8^+^ T cells, and M1 macrophages but decreased the proportion of M2 macrophages in lung tumors (Fig. 7E and F and Fig. S16), with no comparable changes in spleen immune populations (Fig. S17). The TME of *Timeless* knockdown tumors under combination therapy was characterized by elevated ferroptosis (4HNE^+^), increased anti-tumor immunity (CD4^+^ T cells, CD8^+^ T cells, and CD86^+^ macrophages), and decreased protumor M2 macrophages (CD206^+^) (Fig. 7G and Fig. S18). Collectively, these findings suggested that TIMELESS down-regulation potentiates the efficacy of the erastin and anti-PD-1 combination therapy by promoting ferroptosis and remodeling the tumor immune microenvironment.

### High TIMELESS expression in human LUAD is associated with PD-L1 up-regulation, ferroptosis suppression, and altered immune infiltration

**Fig. 8.**
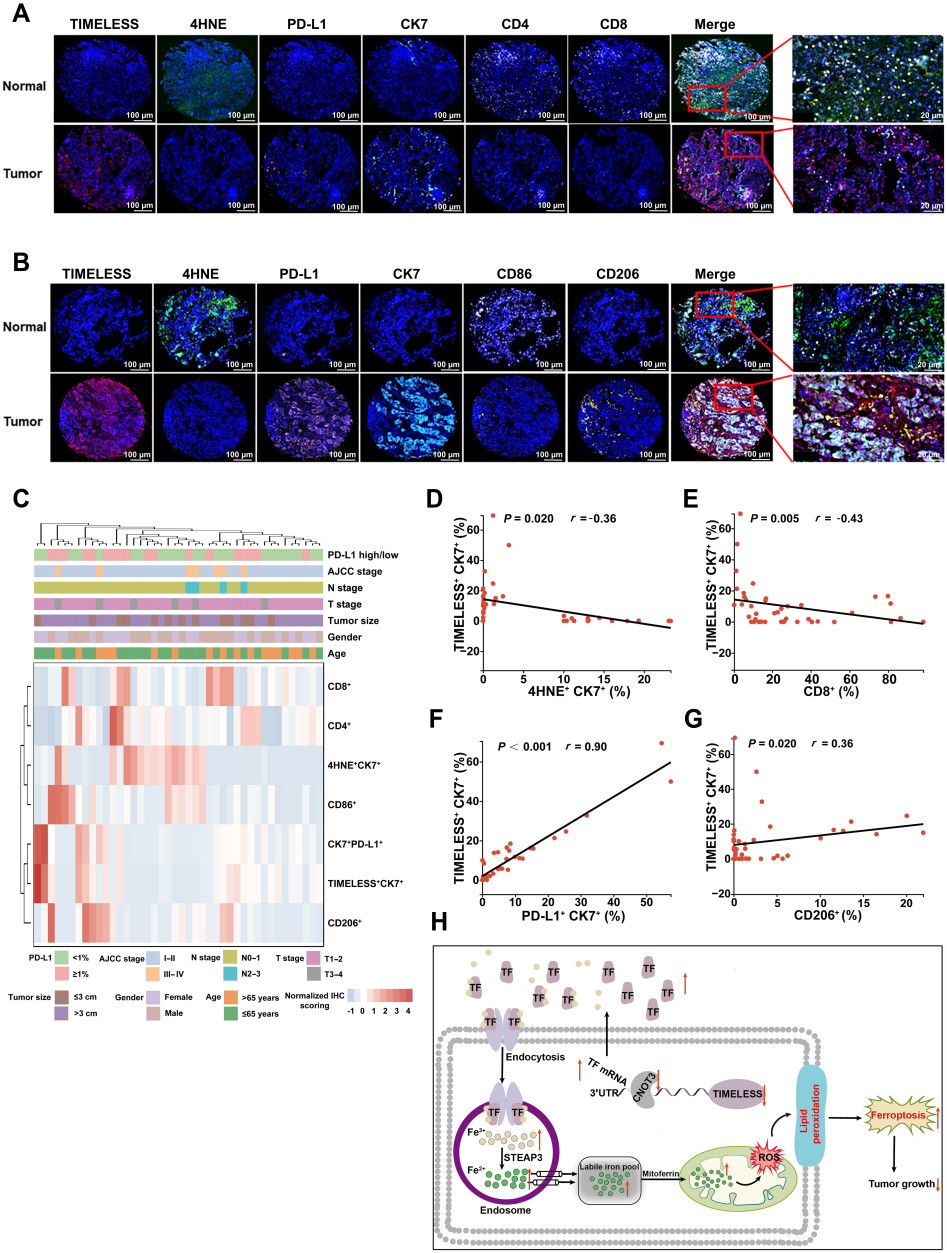
TIMELESS is correlated with ferroptosis, immune infiltration, and disease progression. (A and B) Representative mIF images from 2 independent 6-plex panels in matched normal and tumor tissues of LUAD patient cohort 2 (*n* = 38). Panel 1 stained for TIMELESS (red), 4HNE (green), PD-L1 (orange), CK7 (cyan), CD4 (yellow), and CD8 (white) (A). Panel 2 stained for TIMELESS (red), 4HNE (green), PD-L1 (orange), CK7 (cyan), CD86 (white), and CD206 (yellow) (B). Nuclei were counterstained with DAPI (blue), and TIMELESS, 4HNE, PD-L1, and CK7 served as internal spatial anchors for image alignment and comparative analysis in both panels. (C) Heatmap depicting standardized IHC scores for marker proteins in tumor tissues from LUAD patient cohort 2 (*n* = 42). Each cell in the matrix represents the expression level of a single protein (rows) in an individual sample (columns). (D to G) Correlation analysis of cancer cell TIMELESS expression across the tumor tissues from LUAD cohort 2 (*n* = 42). TIMELESS expression was assessed for correlation with 4HNE expression (D), the extent of CD8^+^ T cell infiltration (E), PD-L1 expression on cancer cells (F), and the density of CD206^+^ M2 macrophage infiltration (G). All markers were assessed by mIF and quantified as the percentage of positive cells. (H) Schematic of the hypothesized mechanism. The model proposes that *TIMELESS* knockdown, through disrupting CNOT3 binding and subsequently increasing TF expression, may drive ferroptosis and immune microenvironment remodeling to suppress tumor growth. The statistical analysis was performed using Spearman rank correlation (D to G). Abbreviations: 4HNE, 4-hydroxynonenal; CD, cluster of differentiation; CNOT3, Ccr4-Not transcription complex subunit 3; CK7, cytokeratin 7; IHC, immunohistochemistry; LUAD, lung adenocarcinoma; mIF, multiplex immunofluorescence; PD-L1, programmed cell death ligand 1; TF, transferrin.

To clinically assess the role of TIMELESS in ferroptosis and immune evasion, we performed mIF on a TMA comprising paired tumor and adjacent normal tissues from 38 patients with LUAD. TIMELESS was predominantly coexpressed with CK7 in tumor cells, and this coexpression was associated with up-regulation of PD-L1 and suppression of the ferroptosis marker 4HNE. High TIMELESS expression was linked to a profoundly immunosuppressive landscape, marked by diminished infiltration of CD4^+^ T cells, CD8^+^ T cells, and CD86^+^ M1 macrophages, with concomitant enrichment of CD206^+^ M2 macrophages (Fig. 8A and B and Fig. S19A). Notably, there was a significant concordance between PD-L1 mRNA and protein levels, with tumors exhibiting high TIMELESS protein levels being significantly more likely to have elevated PD-L1 RNA (Fig. S19B and C). To statistically validate these relationships, hierarchical cluster analysis identified distinct expression patterns across tumor T stages for CD8^+^ T cell density, TIMELESS^+^CK7^+^ cancer cells, and 4HNE^+^CK7^+^ cells, while CD4^+^ T cell infiltration was associated with both PD-L1 high/low groups and T stage progression (Fig. 8C and Fig. S19D). Moreover, TIMELESS expression exhibited significant inverse correlations with 4HNE levels and CD8^+^ T cell infiltration and positive correlations with PD-L1 protein level and CD206^+^ M2 macrophage infiltration (Fig. 8D to G). Although inverse correlation trends of TIMELESS with CD4^+^ T cell and CD86^+^ M1 macrophage infiltration were observed, they did not reach statistical significance (Fig. S19E and F). Among 42 LUAD patients with available follow-up data, those with elevated expression levels of TIMELESS and PD-L1 had poorer survival (Fig. S19G and H). Taken together, these findings suggested that high levels of TIMELESS in LUAD are associated with enhanced PD-L1 expression, reduced levels of 4HNE, decreased infiltration of T cells and M1 macrophages, and increased presence of M2 macrophages.

## Discussion

RBPs are frequently dysregulated in LUAD, contributing to its post-transcriptional landscape [53]. Our analysis of TCGA-LUAD and multiple GEO datasets revealed a widespread pattern of RBPs dysregulation in this malignancy, with *TIMELESS* identified as a significantly up-regulated RBP exhibiting potential oncogenic function. Functional screening and validation revealed that *TIMELESS* knockdown significantly inhibited the growth and viability of LUAD cells. In patient samples, TIMELESS expression was elevated in LUAD tumor tissues compared with normal tissues and was associated with larger tumor size and more advanced disease stage. Although TIMELESS is recognized as a circadian rhythm-associated gene [54], the tumor-specific functions of TIMELESS remain poorly understood. While recent studies have begun to elucidate the oncogenic roles of TIMELESS in other cancers [55,56], its mechanistic contributions to LUAD pathogenesis require further investigation.

Cell death is a fundamental biological process for maintaining cellular homeostasis, regulating tissue development, and modulating disease progression [57]. While oxidative stress is a common feature in many forms of cell death [58], ferroptosis is specifically distinguished by its dependence on iron and the accumulation of lipid peroxides [27]. This regulated cell death form is driven by iron accumulation, ROS generation, and lipid peroxidation of the cell membrane [45]. Emerging research has shown that the induction of ferroptosis suppresses tumor growth, enhances the response to immunotherapy, and helps overcome drug resistance in refractory cancers [59]. Studies have reported that targeting CPT1A augments immune checkpoint blockade (ICB)-induced tumor ferroptosis and anti-tumor immunity in tumor-bearing mice [13]. Furthermore, Yang et al. [42] revealed the heterogeneity of ferroptosis in triple-negative breast cancer, identifying the combination of GPX4 inhibition with ICIs as a promising therapeutic strategy. The present study discovered that *TIMELESS* knockout induced LUAD cell death. Comprehensive analysis characterized the cell death in *TIMELESS* knockdown LUAD cells and mouse models as ferroptosis, as evidenced by significant accumulation of lipid peroxides and intracellular iron, both hallmark features of ferroptosis [60]. Additionally, characteristic mitochondrial shrinkage, a morphological indicator of ferroptosis, was consistently observed. These findings suggested that TIMELESS may be associated with ferroptosis suppression and tumor growth in LUAD, highlighting its potential as a therapeutic target.

The clock protein TIMELESS plays a multifaceted role in cellular homeostasis and tumorigenesis. TIMELESS mitigates replication stress, preserves genomic integrity, and contributes to tumor progression [29]. TIMELESS forms a protective complex with poly(ADP-ribose) polymerase 1 (PARP1) and TIMELESS interacting protein (TIPIN) to safeguard early S-phase replication [30,61]. TIMELESS-mediated circadian rhythm disruption also reprograms cellular metabolism and aberrantly activates signaling pathways, reshaping the TME cells [30]. Moreover, TIMELESS exhibits context-dependent functional polymorphism. In advanced colorectal cancer, TIMELESS overexpression drives primary tumor progression via the TIMELESS/myosin heavy chain 9 (MYH9)/β-catenin axis, promoting proliferation, invasion, and epithelial–mesenchymal transition (EMT) [62]. Conversely, TIMELESS depletion during metastatic adaptation phases enables metastasis by triggering zinc finger E-box binding homeobox 1 (ZEB1)-mediated EMT to overcome replicative stress [31]. Similarly, in breast cancer, TIMELESS synchronously drives proliferation through specificity protein 1 (SP1)-mediated metabolic reprogramming and immune evasion by enhancing PD-L1 transcription [55,63]. In this study, *TF* mRNA was identified as a specific and direct binding target of TIMELESS, and TF was functionally linked to ferroptosis regulation. TIMELESS down-regulation increased the expression levels of TF. Given the connection between mRNA decay and translation, IP-MS and proteomic analyses were utilized, which identified CNOT3 as the key mediator of *TF* mRNA degradation. CNOT3 serves as a core subunit of the Ccr4-Not complex, an essential cellular machinery that executes mRNA degradation through coordinated deadenylation and decapping processes [50]. The present results elucidated that TIMELESS, functioning as an RBP, recruits CNOT3 to degrade TF mRNA, thereby suppressing ferroptosis (Fig. 8H).

The evolving paradigm of translational oncology has redefined NSCLC therapy, with targeted agents and ICIs substantially improving clinical outcomes. However, advanced-stage NSCLC 5-year survival remains below 20%, underscoring the urgent need for innovative therapeutic combinations. Nivolumab (anti-PD-1) has shown paradigm-shifting efficacy, including in neoadjuvant settings [64,65]. A landmark phase 3 trial has revealed significantly improved event-free survival and pathological complete response rates with neoadjuvant nivolumab plus chemotherapy versus chemotherapy alone in resectable NSCLC [65–68]. Ferroptosis is emerging as a critical regulator of antitumor immunity. Although tumor cell ferroptosis modulates immune cell infiltration, the TME may also suppress immunity through ferroptosis-mediated T cell depletion [60,69]. This complex interplay highlights the therapeutic potential of combining ferroptosis modulation with immunotherapy. The present study utilized multiple methodologies to systematically evaluate the therapeutic potential of TIMELESS inhibition in LUAD. In PDO models, *TIMELESS* knockout markedly enhanced sensitivity to erastin, a ferroptosis inducer. Compared with erastin-treated controls, the orthotopic models revealed that *Timeless* knockdown markedly increased the infiltration of CD4^+^ T cells and CD8^+^ T cells. This immunostimulatory effect was partially attenuated upon treatment with the ferroptosis inhibitor ferrostatin-1, yet residual T cell infiltration remained above baseline. Additionally, ferrostatin-1 increased tumor burden in *Timeless* knockdown models relative to controls, suggesting that ferroptosis contributes to the antitumor immunity triggered by TIMELESS suppression. When combined with erastin and PD-1 blockade, *Timeless* knockdown demonstrated superior tumor suppression and enhanced antitumor immunity compared with monotherapies. Clinical correlation analyses further confirmed that elevated TIMELESS expression is associated with ferroptosis resistance, increased PD-L1 levels, reduced infiltration of CD4^+^ T cells, CD8^+^ T cells, and M1 macrophages, and poor prognosis. The above results suggested that the synergistic combination of *TIMELESS* knockdown and ferroptosis induction may represent a potential strategy to enhance the efficacy of ICB in LUAD.

The present study had several inherent limitations. First, as a conserved oncogenic driver, TIMELESS exhibits tissue-specific functional outputs. This heterogeneity impedes pan-cancer therapeutic strategies and necessitates tissue-specific mechanistic investigations. Moreover, the specific immunomodulation induced by TIMELESS deficiency could not be dissociated from general ferroptosis-induced immune remodeling, as residual immune activation persisted despite ferroptosis inhibition. Finally, the mechanistic role of TIMELESS in TF and CNOT3 subcellular trafficking remains uncharacterized. The observed localization shifts may be attributable to CNOT3 destabilization rather than direct transport regulation. Addressing these limitations may provide further verification of the present findings.

## Conclusion

The present study demonstrated that TIMELESS suppresses ferroptosis and promotes tumorigenesis in LUAD. Mechanistically, *TIMELESS* knockdown disrupts CNOT3 recruitment, a molecular bridge connecting the RNA-binding function of TIMELESS to the degradation machinery of the Ccr4-Not complex, thereby inhibiting TF mRNA decay. This cascade induces intracellular accumulation of lipid peroxides and labile iron pools, triggering ferroptosis and remodeling the LUAD tumor immune microenvironment. Collectively, these findings proposed a regulatory role of TIMELESS in LUAD progression, suggesting that it may serve as a therapeutic target.

## Ethical Approval

This study was approved by the Medical Ethics Committee of the Affiliated Hospital of the Air Force Medical University (No. KY20234063-1), and written informed consent was obtained from all participants. The animal studies were conducted in compliance with protocols approved by the Ethics Committee of the Air Force Medical University (No. 20230930).

## Data Availability

Data, analytical methods, and study materials are available from the corresponding authors upon reasonable request. Proteomics data can be retrieved from the iProX database under accession number PXD07021, and the RNA-seq and PAR-CLIP-seq datasets are available in the National Genomics Data Center under BioProject IDs PRJCA049604 and PRJCA049614.
