# Supplementary material for: TIMELESS Promotes LUAD Growth via Suppressing Transferrin-Mediated Ferroptosis and Reprograms the Tumor Microenvironment against Anti-PD-1 Immunotherapy
Source: Cancer Commun (Lond). 2026 Feb 3;46:0009. doi: 10.34133/cancomm.0009 (PMC12864654; doi:10.34133/cancomm.0009)
Supplement: Supplementary 1 — Tables S1 to S8 Figs. S1 to S19 [file cancomm.0009.f1.zip › CANCOMM-D-25-00338_R1_supplementary_final.pdf]

Supplementary Materials for

TIMELESS promotes LUAD growth via suppressing transferrin-mediated ferroptosis and reprograms the tumor microenvironment against anti-PD-1 immunotherapy

Chenchen Hu[1,†], Feiming Hu[1,†], Changjian Shao[2,†], Yuanli He[1,†],Liping Su[1], Daimei Shi[1], Lingying Yu[1], Yuanjie Sun[1], Jing Wang[3], Xiyang Zhang[3], Hongtao Duan[2], Junqi zhang[1], Yubo Sun[1], Dongbo Jiang[1], Xiaolong Yan[2,*], Shuya Yang[1,3*] and Kun Yang[1,*]

[1]Department of Immunology, Basic Medicine School, Air Force Medical University, Xi'an 710032, Shaanxi, P. R. China.

[2]Department of Thoracic Surgery, Tangdu Hospital, Air Force Medical University, Xi'an 710038, Shaanxi, P. R. China.

[3]Military Medical Innovation Center, Air Force Medical University, Xi'an 710032, Shaanxi, P. R. China.

[†]Chenchen Hu, Feiming Hu, Changjian Shao, and Yuanli He contributed equally to this study.

[*]**Correspondence to:**

Kun Yang

E-mail: yangkunkun@fmmu.edu.cn

Department of Immunology, Basic Medicine School, Air Force Medical University, Xi'an 710032, Shaanxi, P. R. China.

Shuya Yang

E-mail: yangshuxiaoya@163.com

Department of Immunology and Military Medical Innovation Center, Basic Medicine School, Air Force Medical University, Xi'an 710032, Shaanxi, P. R. China.

Xiaolong Yan

E-mail: yanxiaolong@fmmu.edu.cn
Department of Thoracic Surgery, Tangdu Hospital, Air Force Medical University, Xi’an 710038, Shaanxi, P. R. China.

Supplementary Tables

Supplementary Table S1. Clinicopathological characteristics of LUAD patients in cohort 1 (n = 90).

Supplementary Table S2. Clinicopathological characteristics of LUAD patients in cohort 2 (n = 92).

Supplementary Table S3. Compilation of 2,961 RNA-binding proteins curated from published datasets.

Supplementary Table S4. The sequences of siRNAs, sh/sgRNAs, and plasmids used in this study.

Supplementary Table S5. Primer sequences for RT-qPCR.

Supplementary Table S6. Primer sequences used for PAT assays.

Supplementary Table S7. The antibodies used in this study.

Supplementary Table S8. The sequences of Luciferase reporter assay and GST pull-down assay.

Supplementary Figures

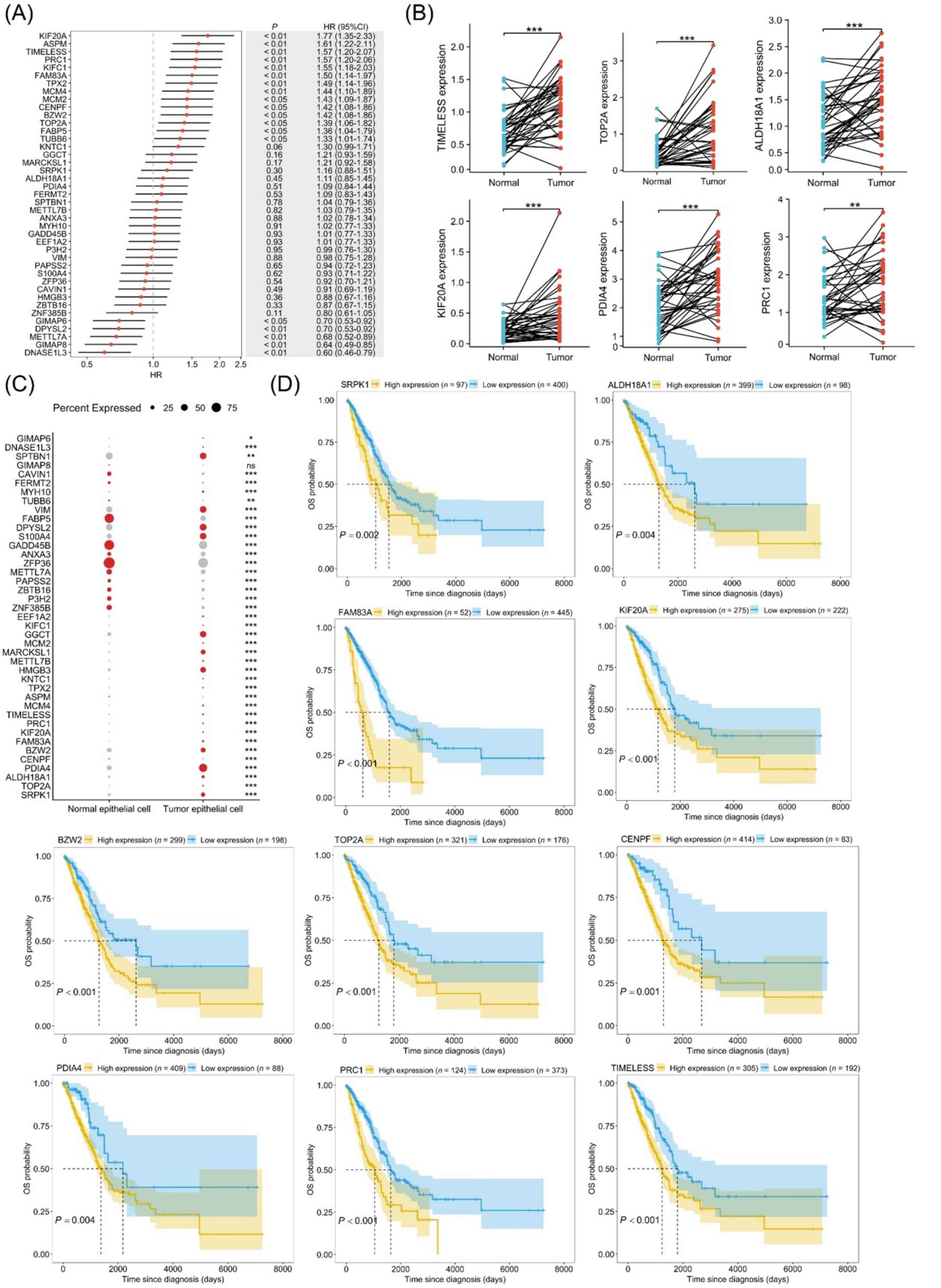

Supplementary Figure S1. The expression characteristics of the differentially expressed RBPs and survival analysis. (A) Forest plot analysis of the effect of RBP genes with low and high expression on the risk of death in TCGA-LUAD patients. **(B)** Expression levels of TIMELESS, TOP2A, ALDH18A1, KIF20A, PDIA4, and PRC1 in LUAD tumor tissues and paired adjacent normal tissues from the GEO dataset (GSE283245; $n = 39$ patient pairs). **(C)** Expression of 41 RBPs in normal and tumor epithelial cells using scRNA-seq data (GSE253013). Gray dots represent low expression,

and red dots represent high expression. **(D)** Kaplan-Meier analysis of OS correlated with the top 10 upregulated RBP genes in the TCGA-LUAD cohort (*n* = 497). Patients were stratified into high and low expression groups based on the optimal cut-point for each gene determined using the "survminer" R package. The statistical analysis was performed using a two-tailed Student's *t*-test (B, C). $*P < 0.05$, $**P < 0.01$, $***P < 0.001$, ns: not significant. Abbreviations: GEO, Gene Expression Omnibus; LUAD, lung adenocarcinoma; OS, overall survival; RBP, RNA-binding protein; scRNA-seq, single-cell RNA sequencing; TCGA-LUAD, The Cancer Genome Atlas-Lung Adenocarcinoma.

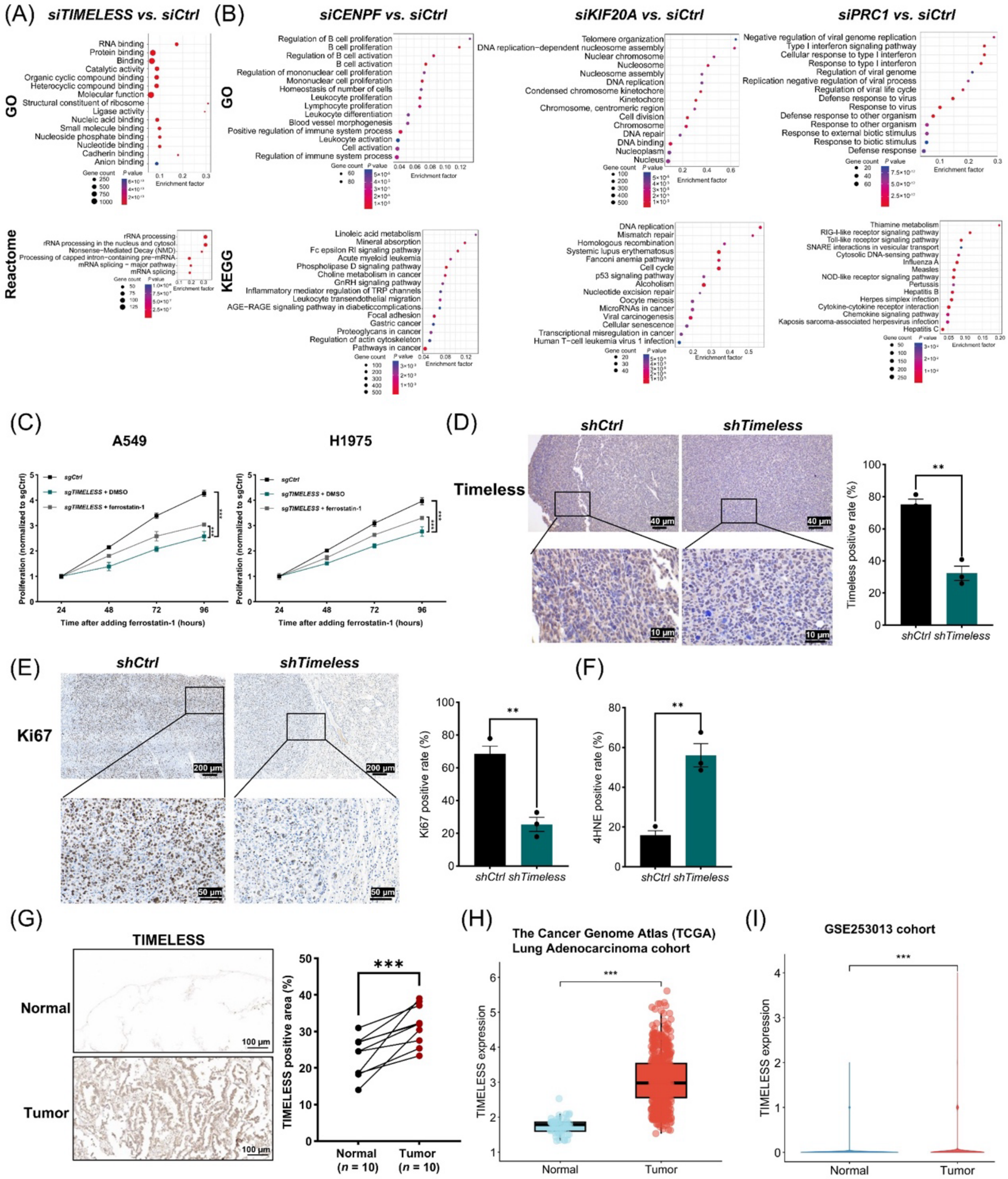

Supplementary Figure S2. Functional enrichment, phenotypic validation, and clinical association of TIMELESS in LUAD. (A) Functional enrichment analysis of DEGs following *TIMELESS* knockdown in H1975 cells: GO biological processes and Reactome pathways. **(B)** GO and KEGG pathway enrichment analysis of DEGs in *CENPF*, *KIF20A,* and *PRC1* knockdown H1975 cells versus control. **(C)** Cell viability of ferrostatin-1-treated *sgTIMELESS* cells was assessed by the CCK-8 assay ($n = 6$). Data were analyzed by two-way ANOVA. **(D, E)** Representative IHC staining images and quantitative analysis of Timeless (D) and Ki67 (E) expressions in the LLC1-induced lung tumor tissues from *shCtrl* and *shTimeless* groups. **(F)** Quantitative analysis of 4HNE expression by IHC staining in LLC1-induced lung tumor tissues from the *shCtrl* and *shTimeless* groups. **(G)** Representative IHC staining image and the corresponding quantification data of TIMELESS expression in LUAD tissues and paired adjacent normal tissues from clinical samples (cohort 3, n =

10 patient pairs). **(H)** TIMELESS expression differences between normal and tumor tissues in TCGA-LUAD (*n* = 58 normal and *n* = 510 tumor tissues). **(I)** TIMELESS expression in normal and tumor epithelial cells from single-cell RNA-seq analysis (GSE253013; *n* = 6 normal vs. *n* = 9 tumor samples). Abbreviations: CCK-8, cell counting kit-8; CENPF, centromere protein F; DEGs, differentially expressed genes; DMSO, dimethyl sulfoxide; GO, Gene Ontology; IHC, immunohistochemistry; KEGG, Kyoto Encyclopedia of Genes and Genomes; KIF20A, kinesin family member 20A; LLC, Lewis lung carcinoma; PI, propidium iodide; PRC1, protein regulator of cytokinesis 1.

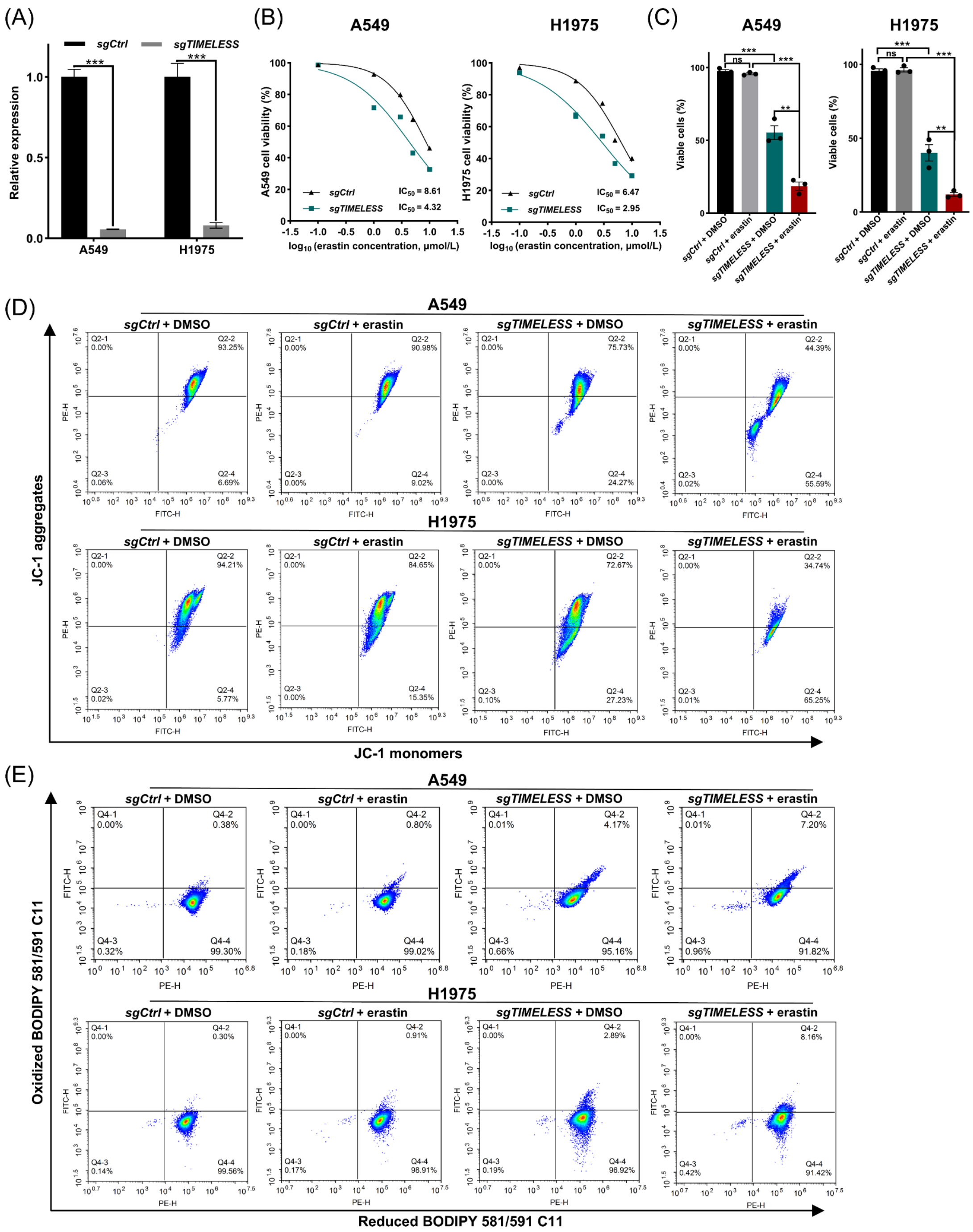

Supplementary Figure S3. Validation of *TIMELESS* knockout efficiency and ferroptosis-related phenotypes: IC_{50} determination, cell viability, MMP and lipid ROS. (A) RT-qPCR showed the efficiency of CRISPR-Cas9-mediated sgRNA knockout of *TIMELESS* in human LUAD cell lines (*n* = 3 per group). **(B)** Determination of erastin (0, 0.1, 1, 3, 5, and 10 μmol/L) IC_{50} after 48-hour treatment between control (*sgCtrl*) and *TIMELESS*-knockout (*sgTIMELESS*) A549 and H1975 cells. **(C)** Quantification of living cells by live/dead staining in *TIMELESS*-knockout and control cells after erastin treatment. **(D)** Flow cytometric analysis of JC-1 staining. The PE-H/FITC-H fluorescence ratio was calculated to reflect the changes of MMP in different groups. **(E)** Flow cytometric analysis of BODIPY 581/591 C11 staining. The PE-H/FITC-H fluorescence ratio was calculated to reflect the

changes of lipid peroxidation. The statistical analysis was performed using a two-tailed Student's *t*-test (A, C). $^{**}P < 0.01$, $^{***}P < 0.001$, ns: not significant. Abbreviations: CRISPR, Clustered Regularly Interspaced Short Palindromic Repeats; IC_{50}, half-maximal inhibitory concentration; MMP, mitochondrial membrane potential; RT-qPCR, reverse transcription quantitative real-time PCR; ROS, reactive oxygen species.

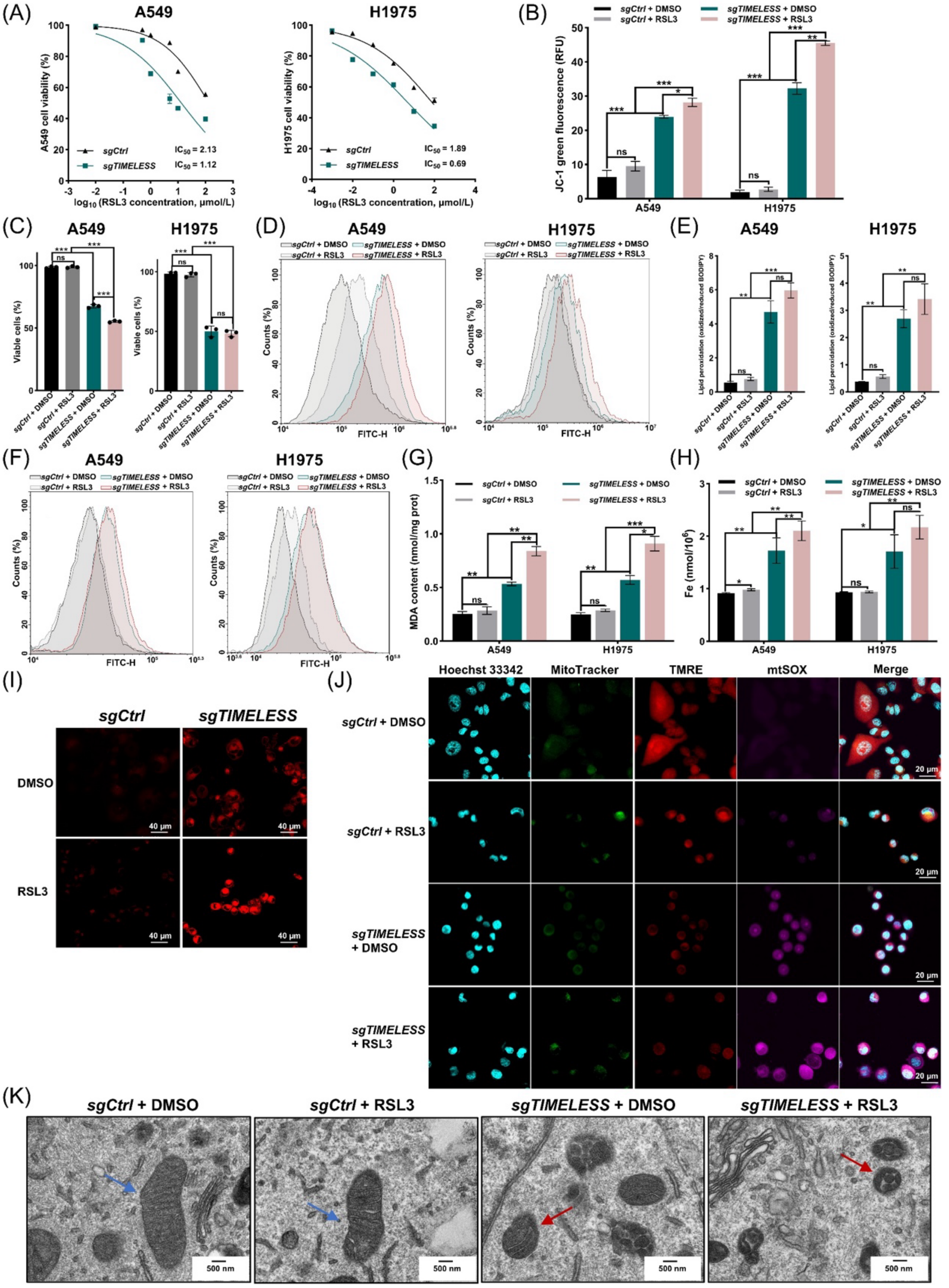

Supplementary Figure S4. TIMELESS deficiency enhances RSL3-induced ferroptosis in LUAD cells. (A) Determination of RSL3 IC_{50} in *TIMELESS*-knockout (*sgTIMELESS*) and control (*sgCtrl*) A549 and H1975 cell lines after 48-hour treatment (0.01, 0.5, 1, 5, 10, 100 μmol/L RSL3). **(B)** MMP was assessed by JC-1 flow cytometry in *sgCtrl* and *sgTIMELESS* A549 and H1975 cells after 48-hour treatment with either DMSO or RSL3 ($n = 3$ per group). **(C)** Quantification of living cells by live/dead staining in *TIMELESS*-knockout and control A549 and H1975 cells after RSL3 treatment. **(D)** Intracellular total ROS were assessed by DCFH-DA fluorescence in A549 and H1975 cells after 48-

hour treatments with DMSO or RSL3. **(E)** Lipid peroxidation were monitored using BODIPY 581/591 C11 staining. Experimental groups were as follows: *sgCtrl* + DMSO, *sgCtrl* + RSL3, *sgTIMELESS* + DMSO, and *sgTIMELESS* + RSL3. **(F)** Lipid peroxides was assessed using the Liperfluo probe and flow cytometry in *sgCtrl* and *sgTIMELESS* cells treated with DMSO or RSL3 for 48 hours. **(G)** MDA content was detected in *sgCtrl* and *sgTIMELESS* cells following DMSO or RSL3 treatment. **(H)** Total iron quantification across indicated groups. **(I)** Labile Fe^{2+} levels were assessed by FerroOrange staining in H1975 cells from the following groups: *sgCtrl* + DMSO, *sgCtrl* + RSL3, *sgTIMELESS* + DMSO, and *sgTIMELESS* + RSL3. **(J)** Multiplexed imaging of nuclei (Hoechst 33342), mitochondrial mass (MitoTracker Green FM), membrane potential (TMRE), and superoxide (mtSOX) in *TIMELESS*-knockout and control cells treated with DMSO or RSL3. **(K)** TEM analysis of mitochondrial ultrastructure in *sgCtrl* and *sgTIMELESS* H1975 cells treated with either DMSO or RSL3. Blue arrows indicated mitochondria with obvious cristae, red arrows indicated shrunken mitochondria. Abbreviations: DCFH-DA, 2’,7’-dichlorodihydrofluorescein diacetate; MDA, malondialdehyde; mtSOX, mitochondrial superoxide; TEM, transmission electron microscopy; TMRE, tetramethylrhodamine ethyl ester.

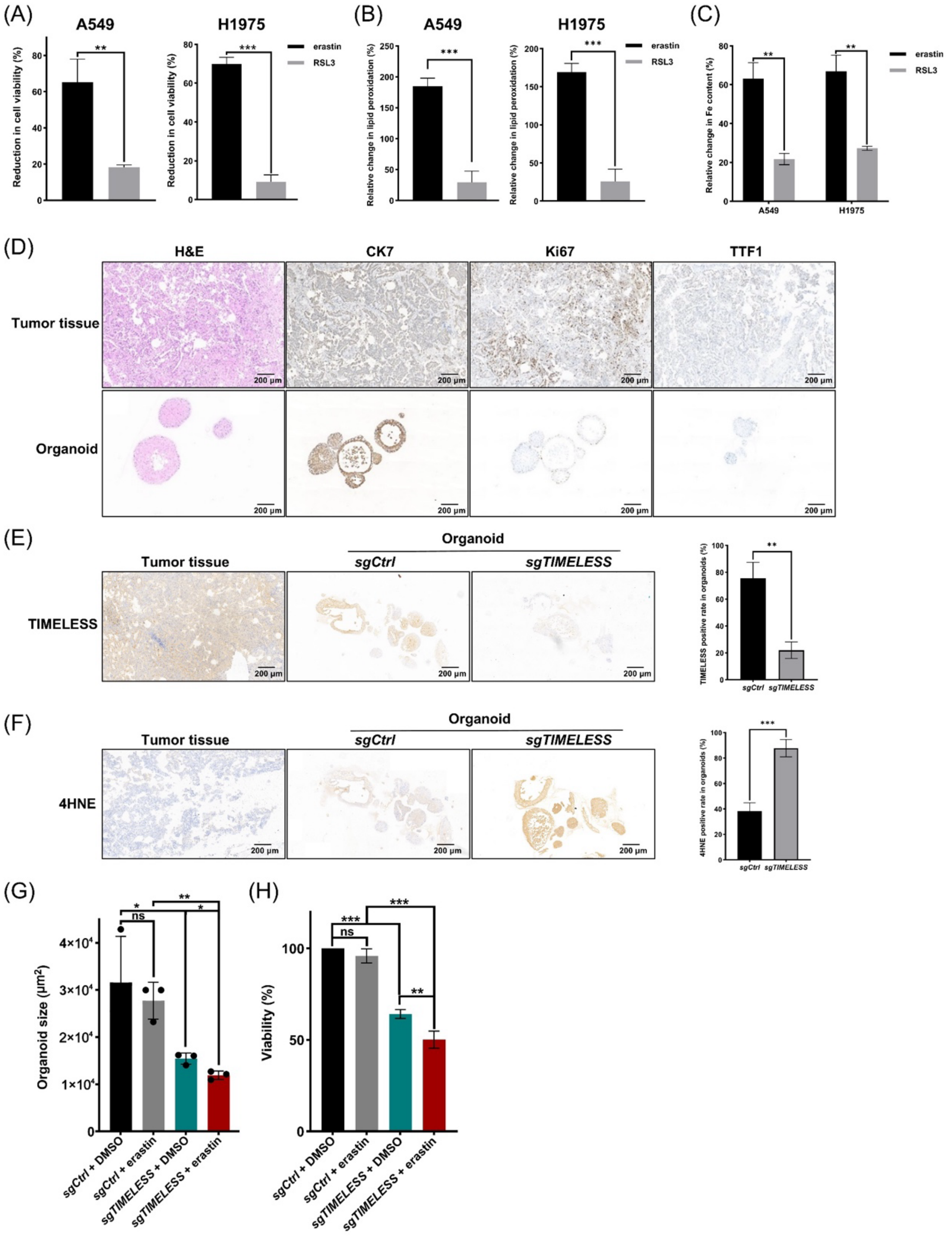

Supplementary Figure S5. Differential effects of ferroptosis inducers erastin and RSL3 on ferroptosis progression in TIMELESS-knockout cells, and suppression of LUAD patient-derived organoid growth by *TIMELESS* knockout. **(A)** Comparison of cell viability reduction induced by erastin versus RSL3. **(B)** Comparison of lipid peroxidation induced by erastin versus RSL3. **(C)** Comparison of intracellular Fe content induced by erastin versus RSL3. **(D)** Representative H&E and IHC staining (CK7, Ki67, and TTF1 markers) images of LUAD patient tumor tissues and the corresponding PDOs. **(E)** Representative IHC staining of TIMELESS in matched primary LUAD

tumor tissue (left panel) and in LUAD PDOs transduced with non-targeting control (*sgCtrl*) or TIMELESS-targeting (*sgTIMELESS*) lentiviruses (middle panel). Quantitative analysis of TIMELESS expression in PDOs is shown on the right (n = 3). **(F)** Representative IHC staining of 4HNE in matched primary LUAD tumor tissue (left panel) and in *sgCtrl* and *sgTIMELESS* LUAD PDOs (middle panel). Quantitative analysis of 4HNE expression in PDOs is shown on the right (n = 3). **(G)** Organoid size was quantified across *sgCtrl* and *sgTIMELESS* LUAD PDOs treated with DMSO or erastin in Matrigel-embedded cultures (n = 3). **(H)** Viability of LUAD PDOs from *sgCtrl* and *sgTIMELESS* groups after treatment with DMSO or erastin. The statistical analysis was performed using a two-tailed Student's t-test (A, B, C, E, F, G, H). *P < 0.05, **P < 0.01, ***P < 0.001, ns: not significant. Abbreviations: 4HNE, 4-hydroxynonenal; DMSO, dimethyl sulfoxide; H&E, hematoxylin and eosin; IHC, immunohistochemistry; LUAD, lung adenocarcinoma; PDOs, patient-derived organoids.

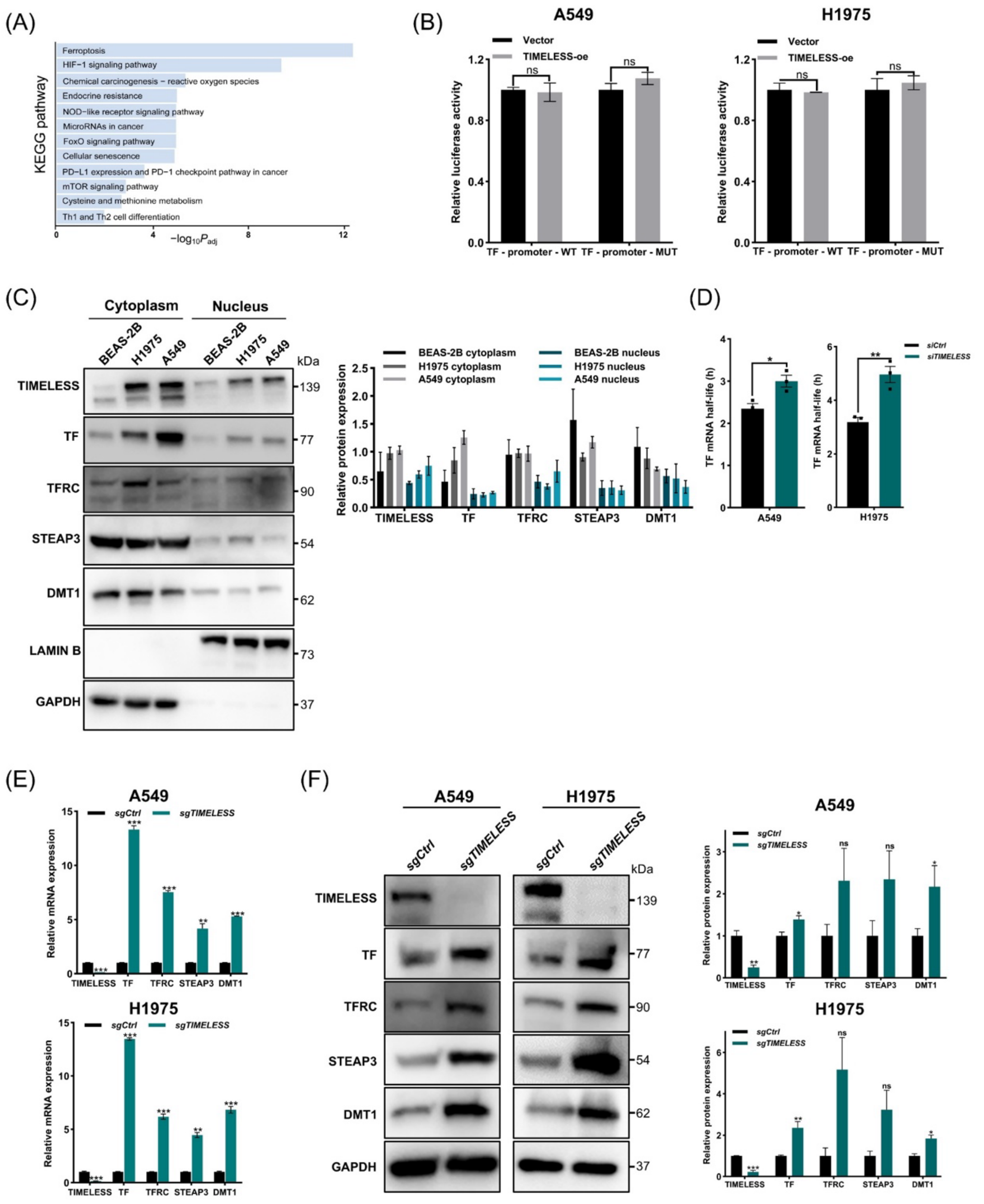

Supplementary Figure S6. TIMELESS negatively regulates iron metabolism-associated genes in LUAD cells. (A) KEGG pathway enrichment of mRNAs directly bound by TIMELESS. **(B)** Relative luciferase activity of the TF promoter following transfection with a control vector or a TIMELESS-overexpressing (TIMELESS-oe) plasmid in A549 and H1975 cells. **(C)** Western blotting analysis of cytoplasmic and nuclear fractions showing the expression of indicated proteins in the human normal bronchial epithelial cell line (BEAS-2B) and LUAD cell lines (H1975 and A549). The band intensity was quantified by ImageJ, and the quantification data are provided in the right. **(D)** TF mRNA half-life in control (*siCtrl*) and *TIMELESS*-knockdown (*siTIMELESS*) LUAD cells. **(E, F)** mRNA and protein expression of iron metabolism-related genes in control (*sgCtrl*) and TIMELESS-knockout (*sgTIMELESS*) cells were analyzed by RT-qPCR (E) and Western blotting (F), respectively. The statistical analysis was performed using a two-tailed Student's *t*-test (B, D, E, F). $*P < 0.05$, $**P <$

0.01, ***$P < 0.001$, ns: not significant. Abbreviations: DMT1, divalent metal transporter 1; STEAP3, six-transmembrane epithelial antigen of prostate 3; TF, transferrin; TFRC, transferrin receptor.

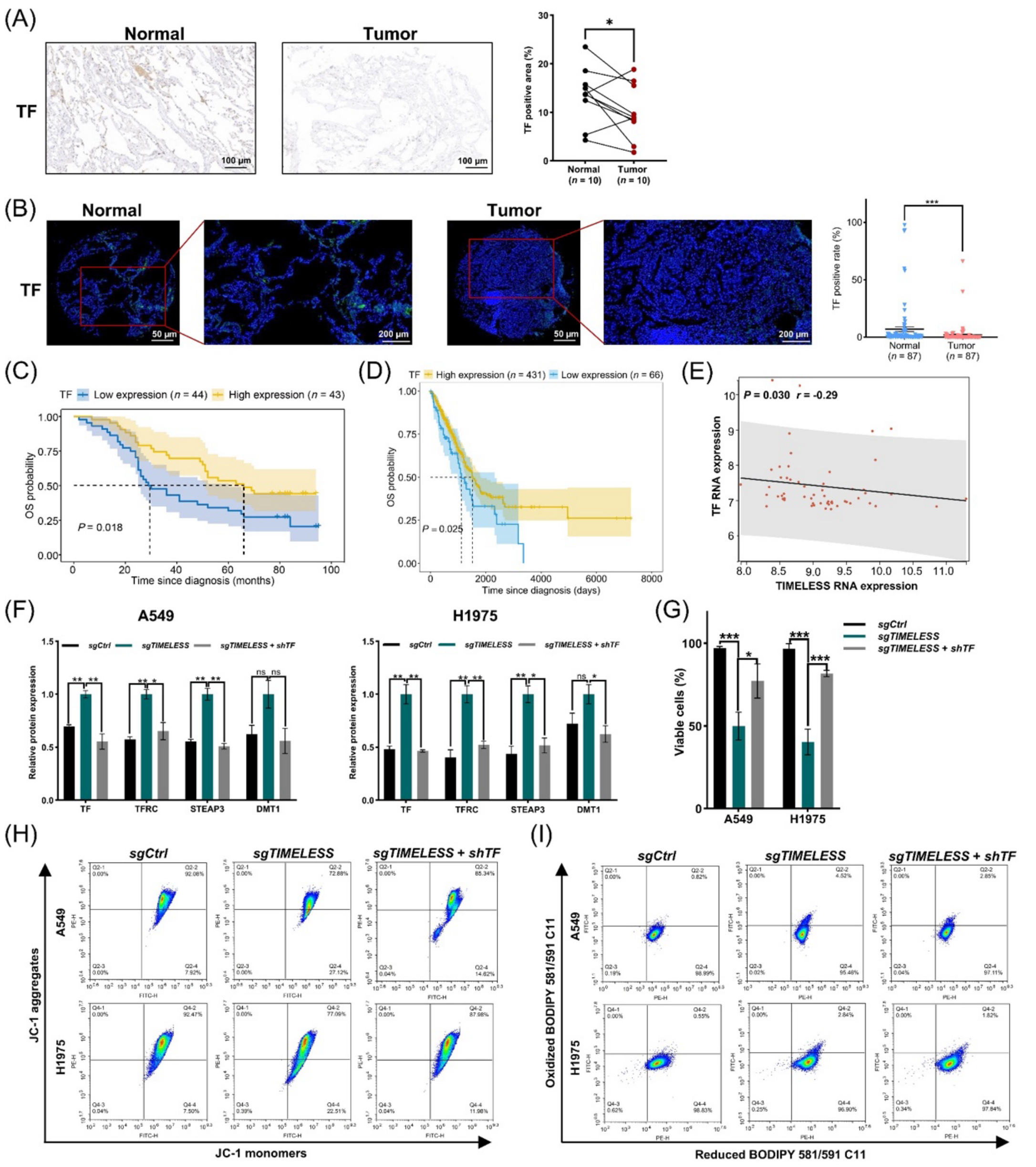

Supplementary Figure S7. Comprehensive analysis of TF in LUAD: integration of expression, survival, molecular profiling, and histopathological validation. (A) Representative IHC staining image and the corresponding quantification data of TF expression in LUAD tissues and paired adjacent normal tissues from clinical samples (n = 10 patient pairs). **(B)** Representative IF images and quantitative analysis of TF expression in tissues from LUAD cohort 1 (n = 87). **(C)** Kaplan-Meier analysis of OS in cohort 1 (n = 87), with patients stratified into high and low groups based on median TF protein expression. **(D)** Kaplan-Meier analysis of OS in the TCGA-LUAD cohort (n = 497): high-versus low-risk groups were stratified by TF mRNA expression with the optimal cut-point determined using the "survminer" R package. **(E)** The correlation between TIMELESS and TF expression was evaluated in LUAD samples from the GSE32863 dataset. **(F)** Quantitative analysis of protein expression by Western blotting in A549 and H1975 cells under the following conditions: control (*sgCtrl*), TIMELESS-knockout (*sgTIMELESS*), and TIMELESS-knockout combined with TF-knockdown (*sgTIMELESS* + *shTF*). **(G)** Live/dead cell staining showed viable cell percentages across the indicated treatment groups. **(H, I)** Representative flow cytometry density plots assessing MMP

(JC-1 staining) (H) and lipid peroxidation levels (BODIPY C11 staining) (I) in the different cell groups. The statistical analysis was performed using a two-tailed Student's *t*-test (A, B, F, G) and Spearman rank correlation (E). Survival curves were plotted by the Kaplan-Meier method (C, D). *$P < 0.05$, **$P < 0.01$, ***$P < 0.001$, ns: not significant. Abbreviations: 4HNE, 4-hydroxynonenal; Fe^{2+}, ferrous iron; IHC, immunohistochemistry; Ki67, kiel antigen 67; LUAD, lung adenocarcinoma; MMP, mitochondrial membrane potential; OS, overall survival; TCGA-LUAD, The Cancer Genome Atlas-Lung Adenocarcinoma; TF, transferrin.

53

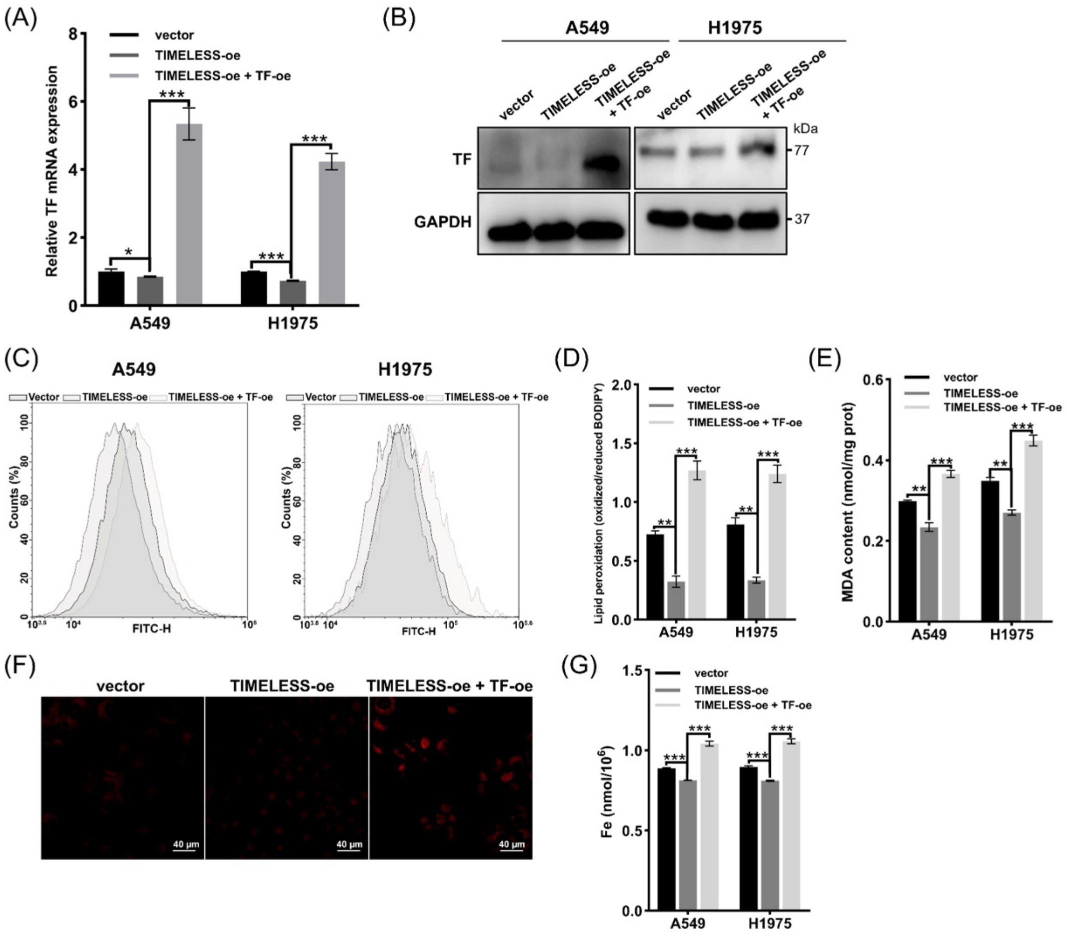

Supplementary Figure S8. Overexpression of TF reverses TIMELESS-mediated ferroptosis resistance in LUAD cells. (A) RT-qPCR analysis of TF mRNA levels in LUAD cells: control (vector), TIMELESS-overexpressing (TIMELESS-oe), and TIMELESS-overexpressing cells with subsequent TF overexpression (TIMELESS-oe + TF-oe). **(B)** TF protein levels in vector, TIMELESS-oe, and TIMELESS-oe + TF-oe LUAD cells, as analyzed by Western blotting. **(C)** Lipid peroxides were detected using the Liperfluo fluorescent probe in vector, TIMELESS-oe, and TIMELESS-oe + TF-oe groups. **(D)** Lipid peroxidation was analyzed using BODIPY 581/591 C11 dye by flow cytometry across the three groups. **(E)** MDA levels were measured in vector, TIMELESS-oe, and TIMELESS-oe + TF-oe LUAD cells. **(F)** Intracellular Fe^{2+} levels were determined by FerroOrange probe staining in the indicated groups. **(G)** Total iron content in vector, TIMELESS-oe, and TIMELESS-oe + TF-oe LUAD cells, as measured by colorimetric assay. The statistical analysis was performed using a two-tailed Student's *t*-test (A, D, E, G). *$P < 0.05$, **$P < 0.01$, ***$P < 0.001$. Abbreviations: TF, transferrin.

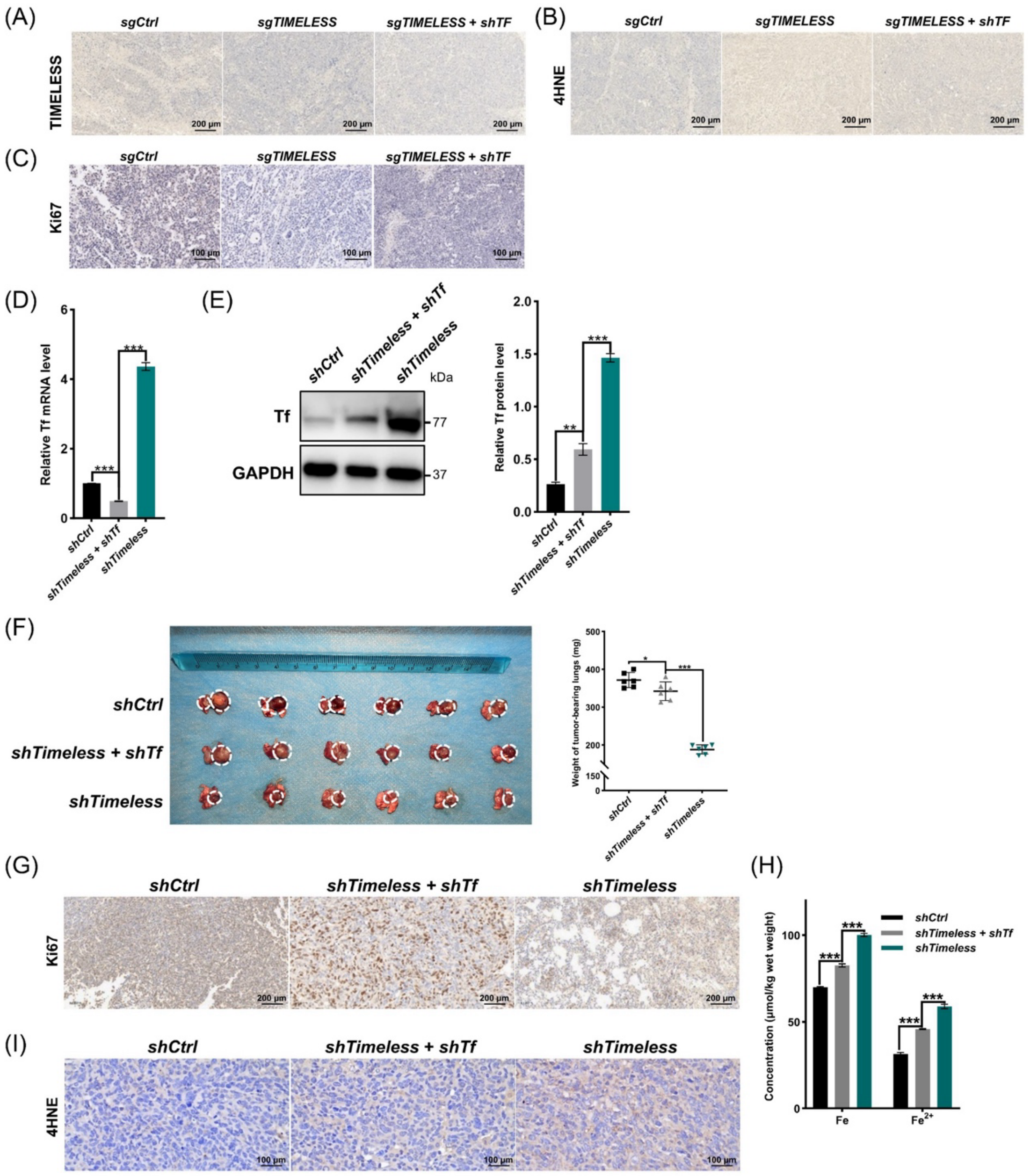

Supplementary Figure S9. *Tf* knockdown rescues tumor growth in *Timeless*-deficient orthotopic LUAD models. (A-C) IHC analysis of xenograft tumors from *sgCtrl*, *sgTIMELESS*, and *sgTIMELESS* + *shTF* groups. Representative images show the expression of TIMELESS (A), the lipid peroxidation marker 4HNE (B), and the proliferation marker Ki67 (C). **(D)** RT-qPCR analysis of *Tf* mRNA levels in mouse LLC1 cells: control (*shCtrl*), *Timeless*-knockdown with subsequent *Tf*-knockdown (*shTimeless* + *shTf*) and *Timeless*-knockdown (*shTimeless*). **(E)** Protein expression was determined by Western blotting in *shCtrl*, *shTimeless* + *shTf*, and *shTimeless* LLC1 cells. **(F)** Weight of tumor-bearing lungs from the indicated groups: *shCtrl*, *shTimeless* + *shTf*, *shTimeless* (n = 6). **(G)** Representative images of Ki67 IHC staining in orthotopic LLC1 lung tumors from the indicated groups. **(H)** Total iron and Fe^{2+} levels in orthotopic tumors across the indicated groups. **(I)** Representative IHC images showing 4HNE expression in LLC1 tumors across the *shCtrl*, *shTimeless* + *shTf*, and *shTimeless* groups. The statistical analysis was performed using a two-tailed Student's t-test (D, E, F, H). $^{*}P < 0.05$, $^{**}P < 0.01$, $^{***}P < 0.001$. Abbreviations: LLC, Lewis lung carcinoma; Tf, transferrin; Ctrl,

control; IHC, immunohistochemistry.

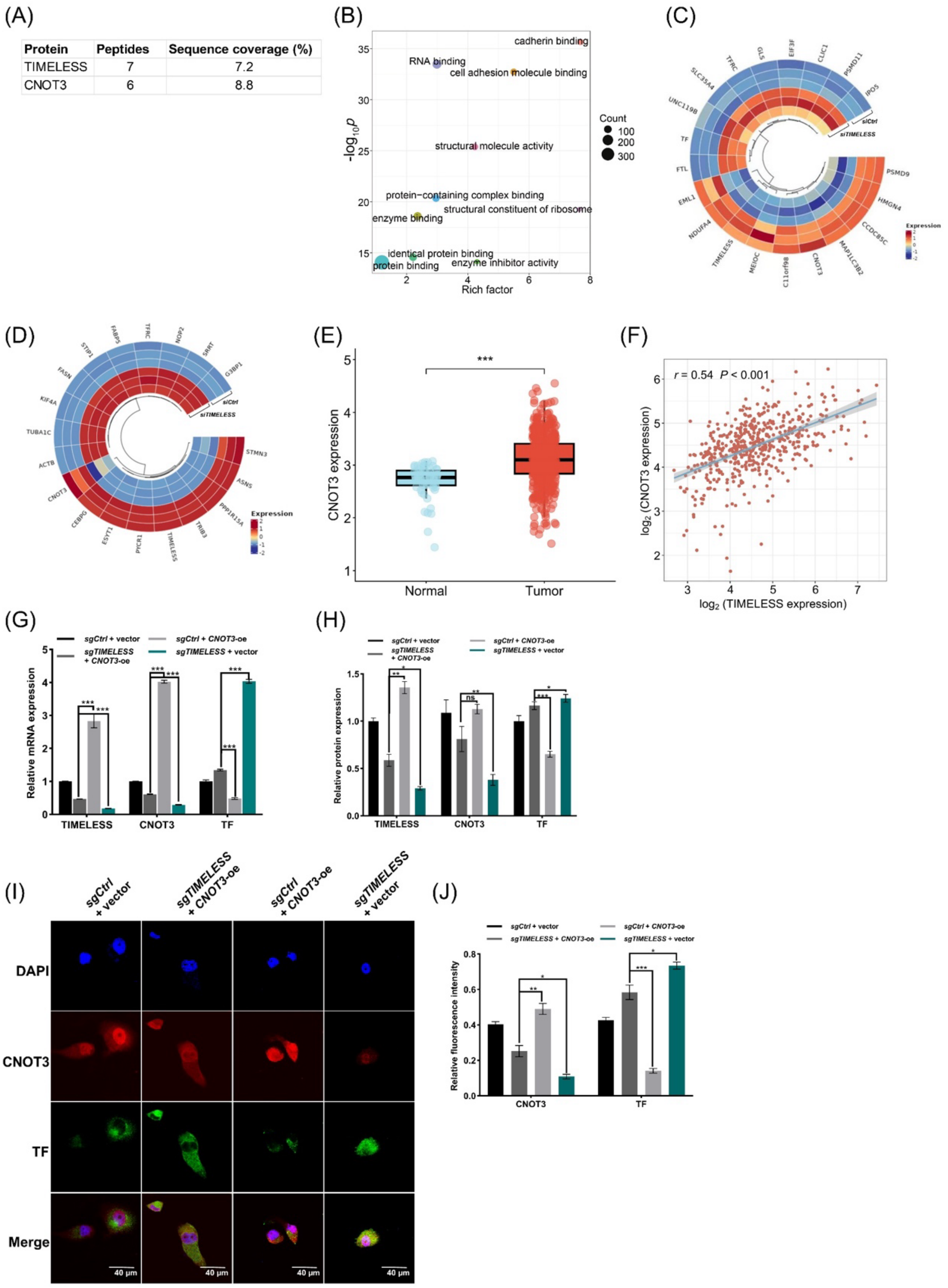

Supplementary Figure S10. The interaction between TIMELESS and CNOT3 and the molecular characteristics of CNOT3 in LUAD. (A) IP-MS identified TIMELESS-CNOT3 interaction. The peptide count and sequence coverage were listed. **(B)** GO enrichment analysis was performed on proteins identified by TIMELESS immunoprecipitation coupled with IP-MS. **(C, D)** Transcriptomic (C) and proteomic (D) profiling of H1975 cells identifies CNOT3 as a downstream protein affected by *TIMELESS* knockdown. **(E)** *CNOT3* mRNA expression in normal and tumor tissues derived from TCGA-LUAD (n = 58 normal and n = 510 tumor tissues). **(F)** Correlation between TIMELESS and CNOT3 expression in tumor samples from the TCGA-LUAD cohort (n = 510 tumor tissues). **(G)** RT-

qPCR analysis of *TIMELESS*, *CNOT3*, and *TF* mRNA levels in H1975 cells across the indicated groups: control of *TIMELESS* knockout + control of *CNOT3* overexpression (*sgCtrl* + vector), *TIMELESS* knockout + *CNOT3* overexpression (*sgTIMELESS* + *CNOT3*-oe), control of *TIMELESS* knockout + *CNOT3* overexpression (*sgCtrl* + *CNOT3*-oe), and *TIMELESS* knockout + control of *CNOT3* overexpression (*sgTIMELESS* + vector). **(H)** Quantitative analysis of Western blotting results for protein expression in *sgCtrl* + vector, *sgTIMELESS* + *CNOT3*-oe, *sgCtrl* + *CNOT3*-oe, and *sgTIMELESS* + vector groups. **(I)** Representative confocal images of CNOT3 and TF in H1975 cells under the indicated conditions: *sgCtrl* + vector, *sgTIMELESS* + *CNOT3*-oe, *sgCtrl* + *CNOT3*-oe, and *sgTIMELESS* + vector. **(J)** Quantitative IF analysis of CNOT3 and TF expression intensity across the indicated groups. The statistical analysis was performed using a two-tailed Student's *t*-test (E, G, H, J) and Spearman rank correlation (F). **P* < 0.05, ***P* < 0.01, ****P* < 0.001, ns: not significant. Abbreviations: CNOT3, Ccr4-Not transcription complex subunit 3; GO, Gene Ontology; IF, immunofluorescence; IP-MS, immunoprecipitation-mass spectrometry; TCGA-LUAD, The Cancer Genome Atlas-lung adenocarcinoma; TF, transferrin.

56

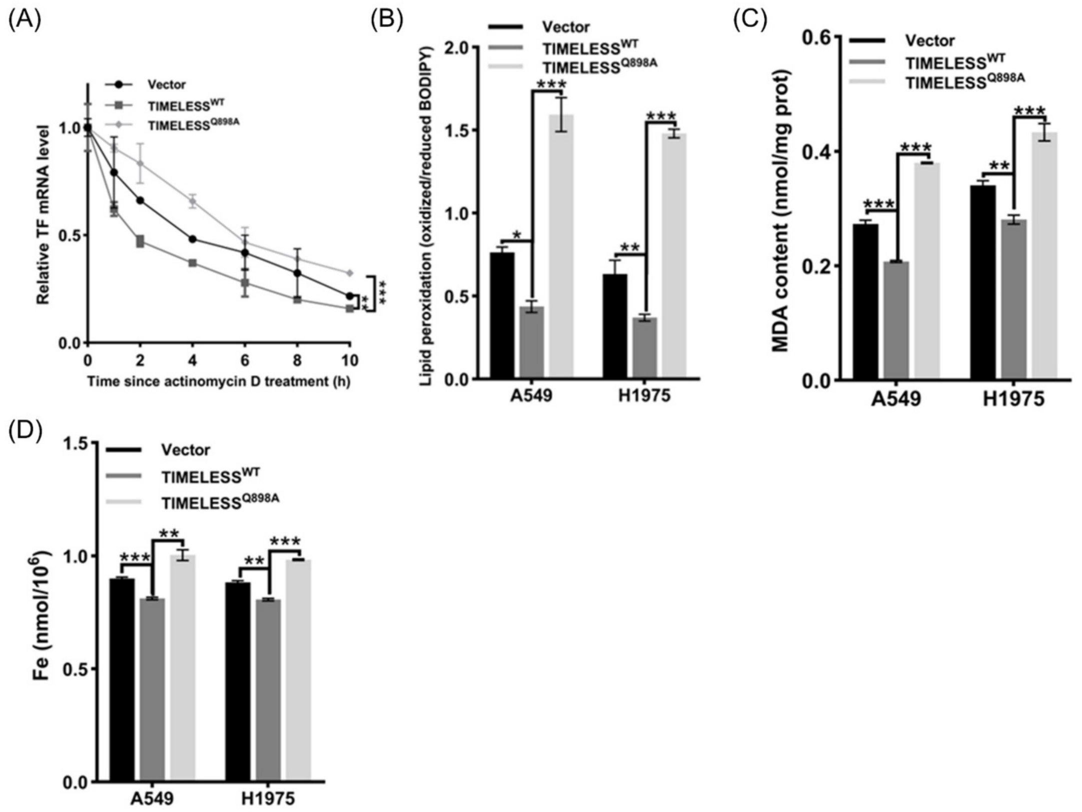

Supplementary Figure S11. TIMELESS-CNOT3 interaction suppresses ferroptosis. (A) TF mRNA stability assessed by actinomycin D assay in H1975 cells transfected with flag-tagged wild-type TIMELESS (TIMELESS^{WT}), the flag-tagged Q898A mutant (TIMELESS^{Q898A}), or empty vector. **(B)** Quantification of lipid peroxidation using BODIPY 581/591 C11 probe across the indicated groups. **(C)** MDA content measured by thiobarbituric acid assay in A549 and H1975 cells expressing TIMELESS^{WT}, TIMELESS^{Q898A}, or an empty vector control. **(D)** Quantification of intracellular Fe levels across the experimental groups. Abbreviations: MDA, malondialdehyde.

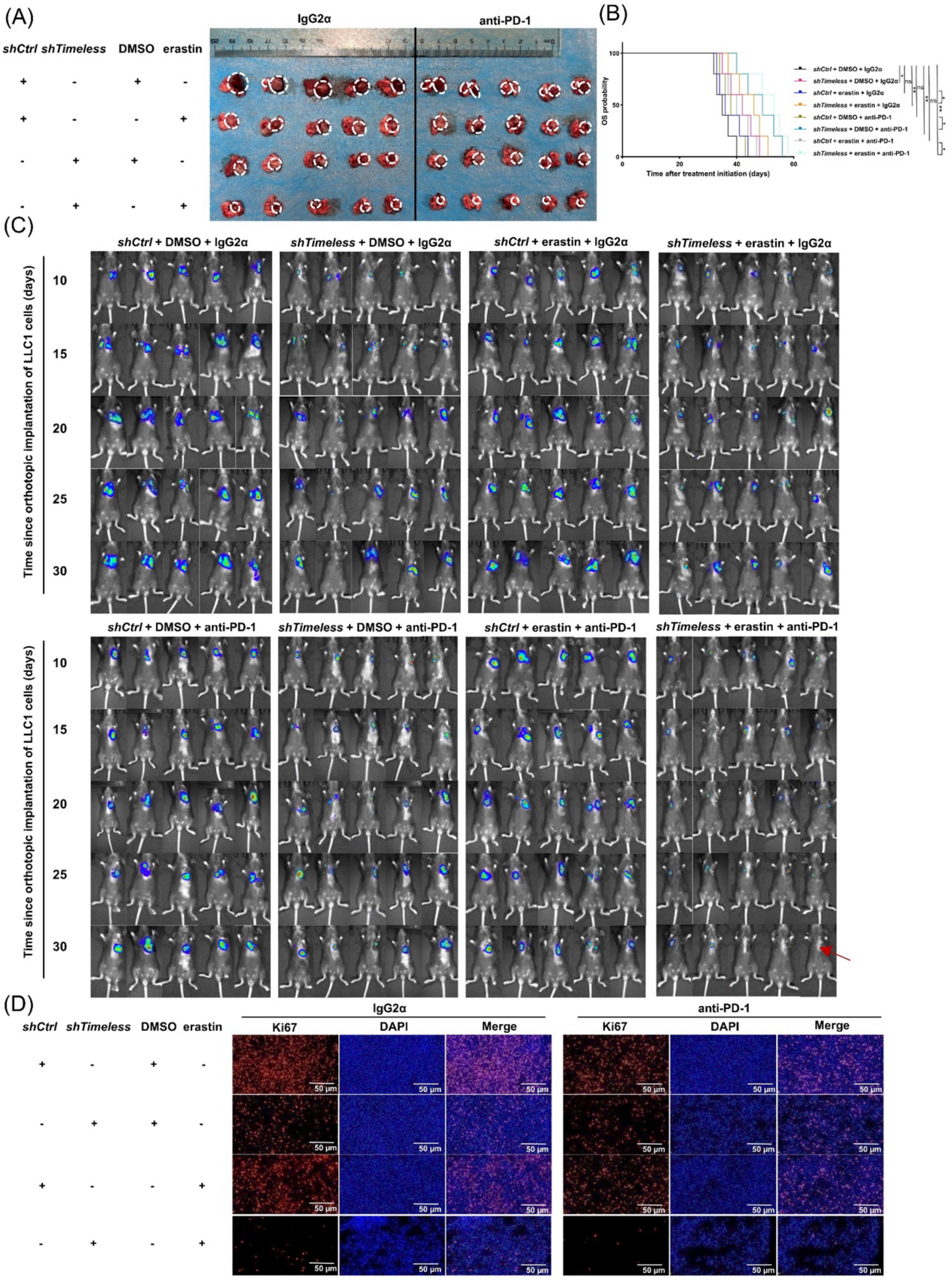

Supplementary Figure S12. Synergistic inhibition of orthotopic lung tumor growth by *Timeless* knockdown combined with erastin and PD-1 blockade. (A) Representative images of orthotopic lung tumors from LLC1 models expressing control shRNA (*shCtrl*) or *Timeless*-targeting shRNA (*shTimeless*), treated with the indicated combinations of DMSO, erastin, IgG2α isotype control, or anti-PD-1 antibody. **(B)** Kaplan-Meier survival curves showing the OS of mice bearing orthotopic LLC1 tumors. The tumors were established using LLC1 cells stably transfected with *shCtrl* or *shTimeless*, and the mice were treated with the indicated regimens. **(C)** In vivo bioluminescence imaging of orthotopic LLC1 tumors at the indicated time points post-treatment initiation across the

experimental groups. Red arrows mark the groups exhibiting pronounced tumor regression. **(D)** Representative IF images of Ki67 staining in sections of orthotopic lung tumors from the indicated treatment groups. The survival time of mice was performed by Kaplan-Meier method (B). *P < 0.05, **P < 0.01, ns: not significant. Abbreviations: OS, overall survival; DMSO, dimethyl sulfoxide; PD-1, programmed cell death protein 1; DAPI, 4',6-diamidino-2-phenylindole dihydrochloride.

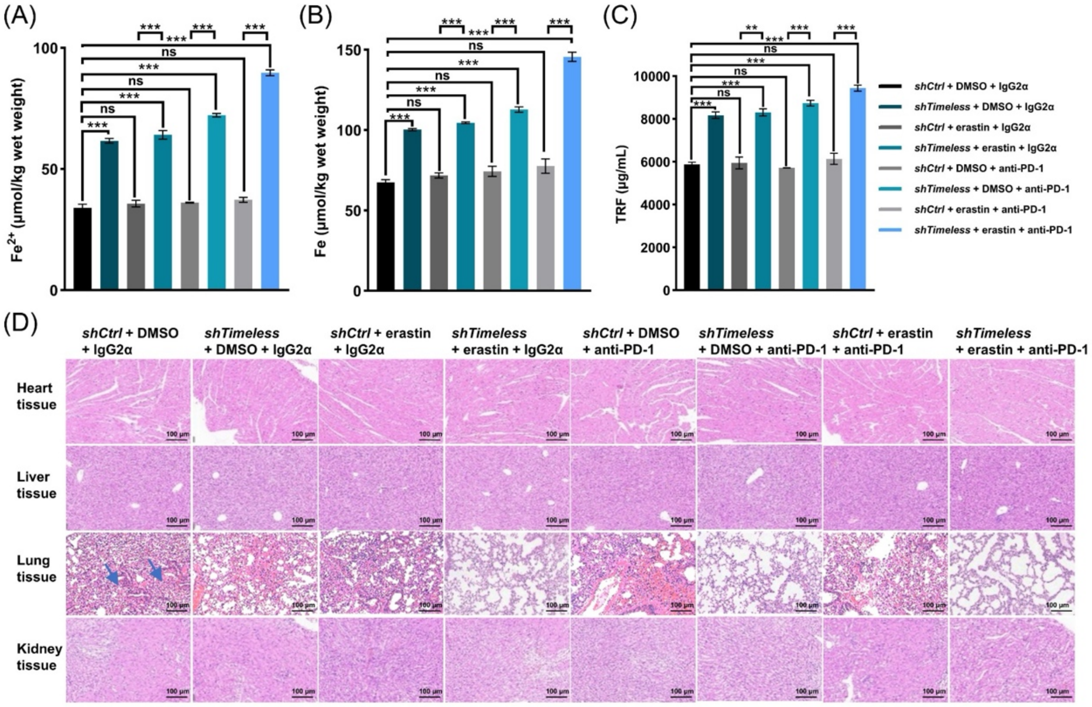

Supplementary Figure S13. Combined erastin and PD-1 blockade treatment induces ferroptosis and provides a favorable safety profile in *Timeless*-knockdown orthotopic lung tumors. (A-C) Fe^{2+} (A), total iron (B), and circulating TF (TRF) levels (C) in orthotopic tumors with or without *Timeless* knockdown (*shTimeless*) in response to combined erastin and PD-1 blockade treatment. Statistical analyses were performed using a two-tailed Student's *t*-test. Primary comparisons were made between each treatment group and the untreated control, with significance indicated above the bars. Further comparisons were conducted between monotherapy and combination therapy groups, as well as between *shTimeless* and *shCtrl* groups. **(D)** H&E staining of heart, liver, lung and kidney tissue sections for toxicity assessment. Arrows highlight pathological features in the control lung tissues. Abbreviations: H&E, hematoxylin and eosin.

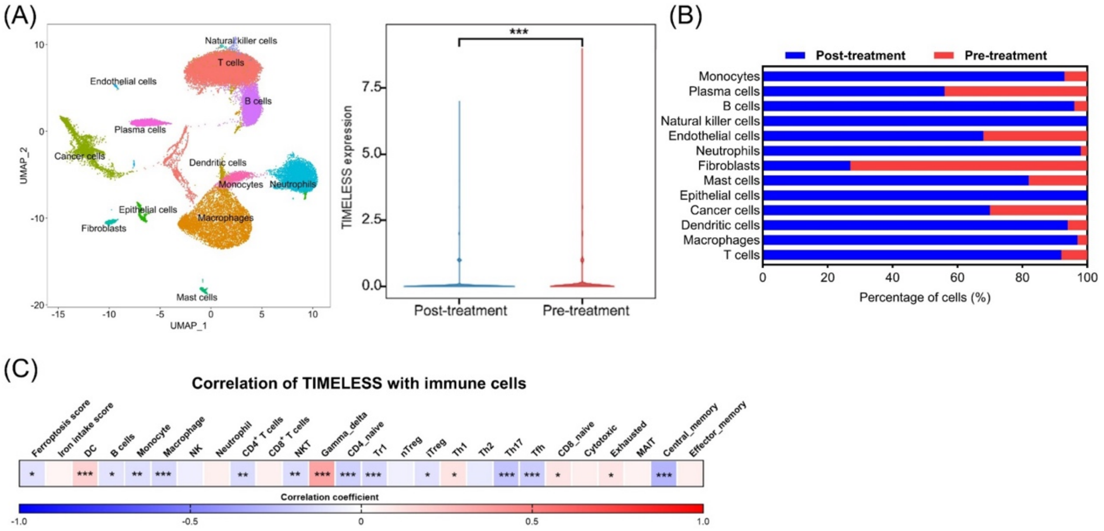

Supplementary Figure S14. Association of TIMELESS with ferroptosis, immune infiltration, and cellular composition in LUAD. (A) scRNA-seq analysis of LUAD patients pre- and post-treatment with anti-PD-1 plus chemotherapy (GSE207422). Left panel: cell type annotation. Right panel: TIMELESS expression levels. **(B)** Compositional shifts in the proportions of indicated cell types in LUAD patients following therapeutic intervention. **(C)** Correlation analysis of TIMELESS expression with ferroptosis score and immune cell infiltration in the TCGA-LUAD cohort (*n* = 510 tumor tissues) using ImmuCellAI. Abbreviations: CD, cluster of differentiation; ImmuCellAI, Immune Cell Abundance Identifier.

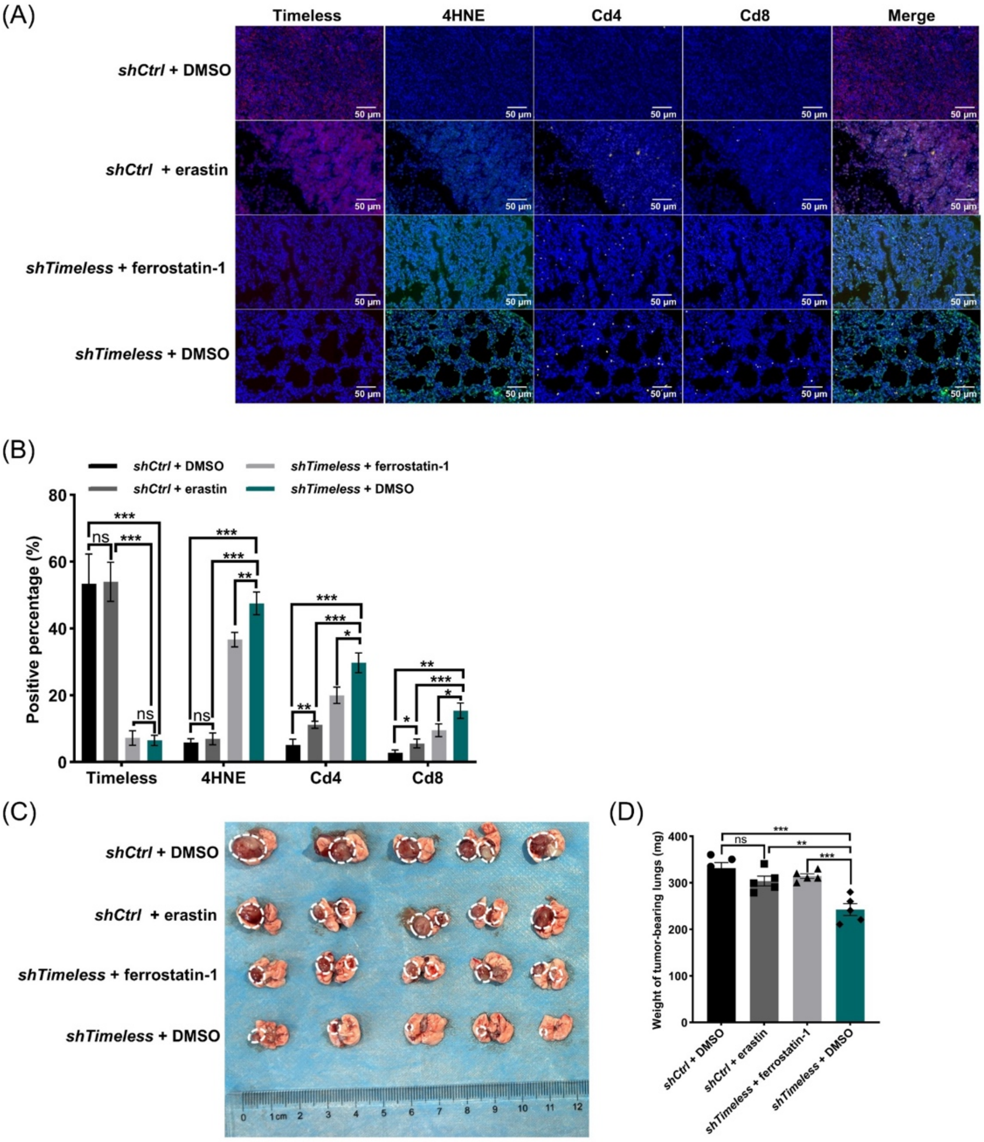

Supplementary Figure S15. Analysis of immune contexture and tumor burden in response to *Timeless* knockdown and ferroptosis modulation. **(A)** Representative mIF staining images of orthotopic lung tumor sections from the indicated experimental groups (*shCtrl* and *shTimeless*: treated with DMSO, erastin, or ferrostatin-1), showing the expression of Timeless (red), 4HNE (green), Cd4 (yellow), and Cd8 (white). Nuclei are counterstained with DAPI (blue). **(B)** mIF quantification of Timeless, 4HNE, Cd4, and Cd8 levels in orthotopic lung tumors from the indicated experimental groups (*shCtrl* and *shTimeless*: treated with DMSO, erastin, or ferrostatin-1). **(C)** Representative images of orthotopic lung tumors from the indicated experimental groups. **(D)** Quantitative analysis of tumor weight across the indicated experimental groups. The statistical analysis was performed using a two-tailed Student's *t*-test (B, D). **P* < 0.05, ***P* < 0.01, ****P* < 0.001; ns: not significant. Abbreviations: 4HNE, 4-hydroxynonenal; DAPI, 4',6-diamidino-2-phenylindole; mIF, multiplex immunofluorescence.

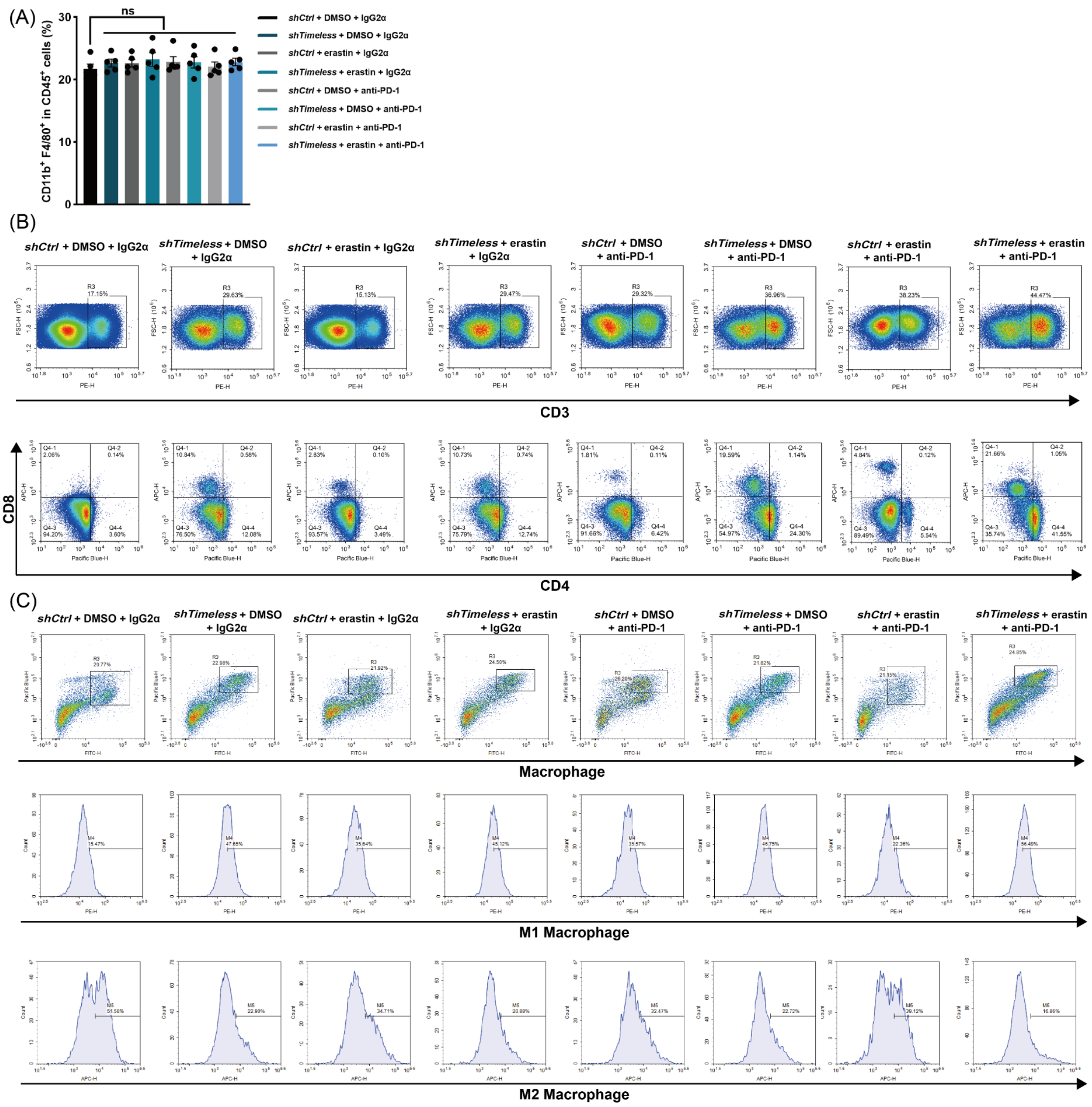

Supplementary Figure S16. Flow cytometry analysis of immune cell populations in orthotopic lung tumors with *Timeless* knockdown under combination therapy. (A) Percentage of CD11b$^+$F4/80$^+$ cells among CD45$^+$ cells in orthotopic lung tumors from LLC1 models expressing control shRNA (*shCtrl*) or *Timeless*-targeting shRNA (*shTimeless*), treated with the indicated combinations of DMSO, erastin, IgG2α isotype control, or anti-PD-1 antibody. Statistical analyses were performed using a two-tailed Student's *t*-test, not significant (ns) indicates no statistically significant differences between the untreated control and the treatment groups. **(B)** Representative flow cytometry density plots for CD3e$^+$, CD4$^+$, and CD8$^+$ T cells in lung orthotopic tumors. **(C)** Representative density plots for macrophages and M1, M2 macrophages in lung orthotopic tumors.

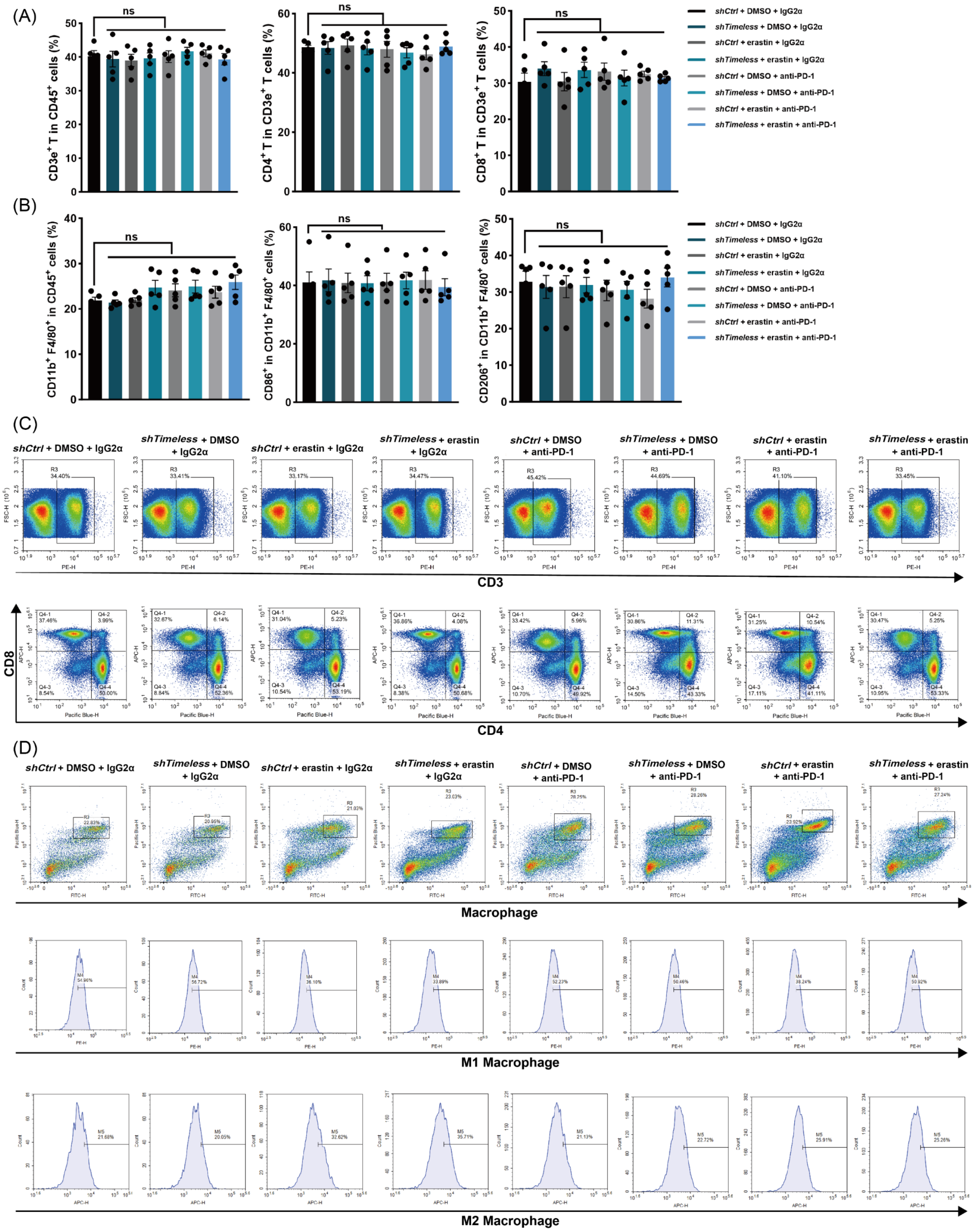

Supplementary Figure S17. Immune cell profiling in the spleens of orthotopic LLC1 models with Timeless modulation under combination therapy. (A) Quantification of T cell populations in spleens from orthotopic LLC1 models (*shCtrl* and *shTimeless* group: treated with DMSO + IgG2α, erastin + IgG2α, DMSO + anti-PD-1, or erastin + anti-PD-1), showing the percentage of CD3e$^+$ T among CD45$^+$ cells, CD4$^+$ T among CD3e$^+$ T cells, CD8$^+$ T among CD3e$^+$ T cells. **(B)** Analysis of macrophage populations in spleens across the indicated groups, including the percentage of CD11b$^+$F4/80$^+$ cells among CD45$^+$ cells, CD86$^+$ cells among CD45$^+$CD11b$^+$F4/80$^+$ cells, and CD206$^+$ cells among CD45$^+$CD11b$^+$F4/80$^+$ cells. **(C)** Representative flow cytometry density plots for CD3e$^+$, CD4$^+$, and CD8$^+$ T cells gating from splenocytes. **(D)** Representative density plots illustrating the

identification of F4/80^{+} macrophages and their CD86^{+} (M1-like) and CD206^{+} (M2-like) subsets in splenic samples. The statistical analysis was performed using a two-tailed Student's *t*-test (A, B), ns: not significant.

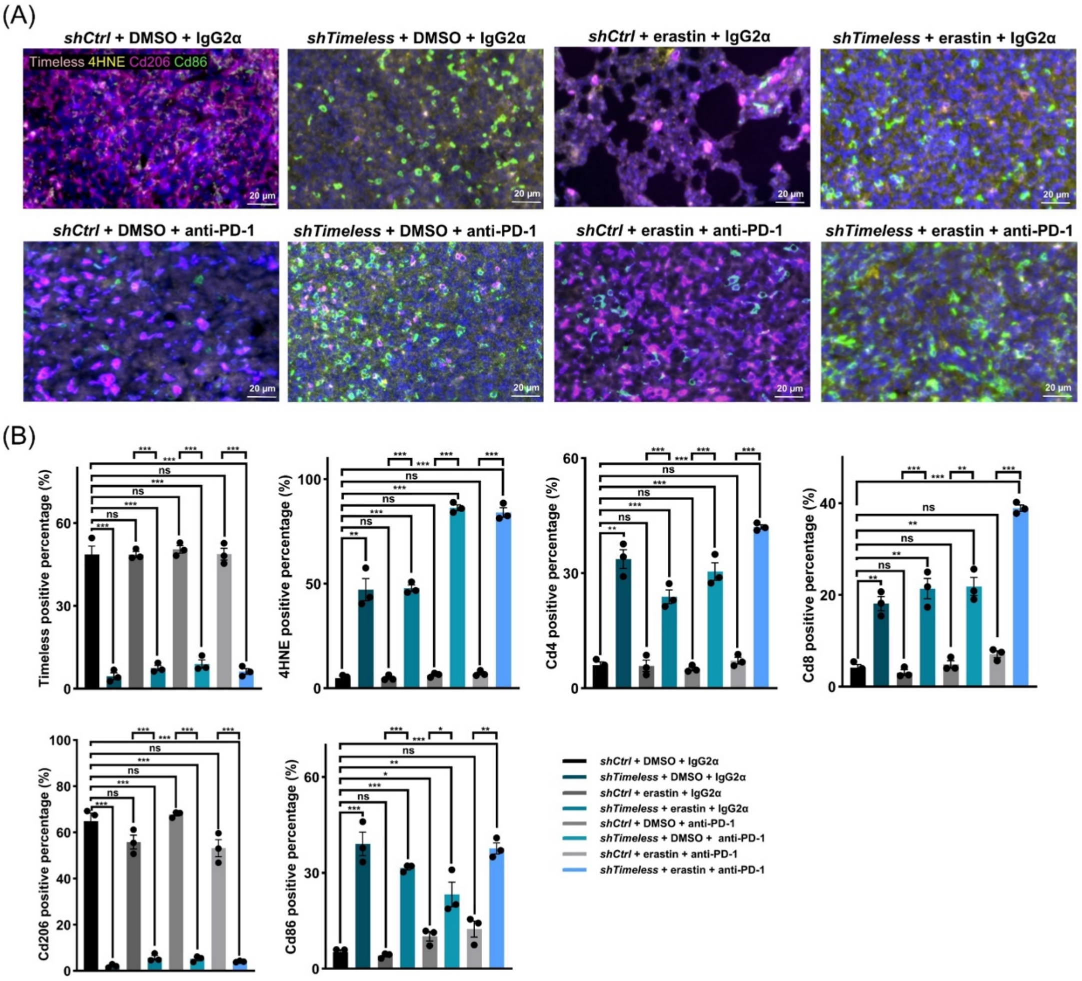

Supplementary Figure S18. Representative images of mIF staining in orthotopic lung tumors from LLC1 models. (A) Representative mIF staining of orthotopic lung tumors for Timeless (pink), 4HNE (yellow), Cd206 (purple), and Cd86 (green) in the indicated groups (*shCtrl* and *shTimeless* group: treated with DMSO + IgG2α, erastin + IgG2α, DMSO + anti-PD-1, or erastin + anti-PD-1). Nuclei are counterstained with DAPI (blue). **(B)** Quantitative analysis of Timeless, 4HNE, Cd4, Cd8, Cd206, and Cd86 expression under different treatments. Abbreviations: 4HNE, 4-hydroxynonenal; DMSO, dimethyl sulfoxide; mIF, multiplex immunofluorescence; PD-1, programmed cell death protein 1.

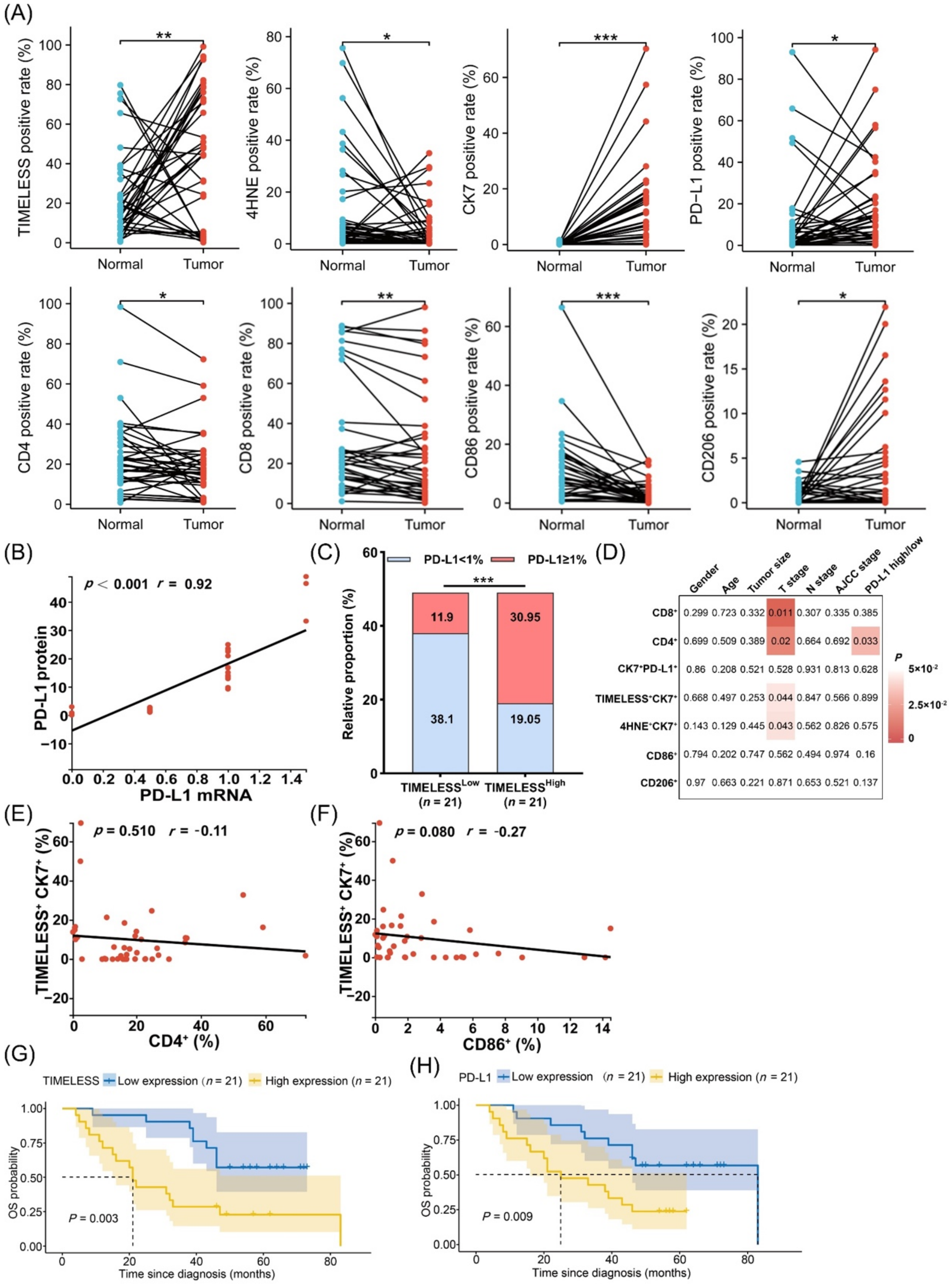

Supplementary Figure S19. High TIMELESS expression in cancer cells of LUAD clinical samples is associated with increased PD-L1, reduced infiltration of $CD4^+$ T cells and M1 macrophages, and poor prognosis. (A) Quantitative analysis of mIF data presented in Figure 8A and B, measuring protein levels of TIMELESS, 4HNE, CK7, PD-L1, CD4, CD8, CD86, and CD206 in LUAD cohort 2 (*n* = 38, paired tumor and normal samples). **(B)** Correlation between PD-L1 mRNA and protein expression in tumor tissue from the LUAD cohort 2 (*n* = 42, tumor samples). **(C)** Association between TIMELESS protein expression and PD-L1 mRNA expression was analyzed. Patients were grouped into PD-L1 positive (≥1%) and negative (<1%) based on the percentage of

stained tumor cells. High and low TIMELESS expression groups were defined by the median gene expression value. **(D)** Statistical analysis of differential gene expressions in Figure 8C. Gene expression levels were compared across gender, age, tumor size, T and N stages, AJCC stage, and PD-L1 expression. **(E, F)** Correlation between cancer cell TIMELESS expression and CD4$^+$ T cell (E) and CD86$^+$ M1 macrophages (F) infiltration in LUAD cohort 2 (n = 42, tumor samples). All markers were assessed by mIF and quantified as the percentage of positive cells. **(G, H)** Kaplan-Meier analysis of OS stratified by the median expression of TIMELESS (G) and PD-L1 (H) in the LUAD cohort 2 (n = 42, tumor samples). The statistical analysis was performed using paired t-test (A), Chi-square test (C), Wilcoxon Mann-Whitney test (D) and Spearman rank correlation (B, E, F). The survival time of LUAD patients was performed by Kaplan-Meier method (G, H). *$P < 0.05$, **$P < 0.01$, ***$P < 0.001$.

64
